# Predicting an unstable tear film through artificial intelligence

**DOI:** 10.1038/s41598-022-25821-y

**Published:** 2022-12-10

**Authors:** Fredrik Fineide, Andrea Marheim Storås, Xiangjun Chen, Morten S. Magnø, Anis Yazidi, Michael A. Riegler, Tor Paaske Utheim

**Affiliations:** 1 Department of Medical Biochemistry, Oslo University Hospital, Oslo, Norway; 2 The Norwegian Dry Eye Clinic, Ole Vigs Gate 32 E, 0366 Oslo, Norway; 3 Department of Holistic Systems, SimulaMet, Oslo, Norway; 4 Department of Computer Science, Faculty of Technology, Art and Design, Oslo Metropolitan University, Oslo, Norway; 5 University of Tromsø, The Arctic University of Norway, Tromsø, Norway; 6 Department of Computer Science, NTNU, Norwegian University of Science and Technology, Trondheim, Norway; 7 Department of Ophthalmology, Sørlandet Hospital Arendal, Arendal, Norway; 8 Department of Ophthalmology, Vestre Viken Hospital Trust, Drammen, Norway; 9 Department of Oral Surgery and Oral Medicine, Faculty of Dentistry, University of Oslo, Oslo, Norway; 10 Department of Plastic and Reconstructive Surgery, Oslo University Hospital, Oslo, Norway; 11 Department of Ophthalmology, University of Groningen, University Medical Center Groningen, Groningen, The Netherlands; 12 Institute of Clinical Medicine, Faculty of Medicine, University of Oslo, Oslo, Norway; 13 Department of Ophthalmology, Vestfold Hospital Trust, Tønsberg, Norway; 14 Department of Ophthalmology, Stavanger University Hospital, Stavanger, Norway; 15 Department of Clinical Medicine, Faculty of Medicine, University of Bergen, Bergen, Norway; 16 Department of Quality and Health Technology, The Faculty of Health Sciences, University of Stavanger, Stavanger, Norway; 17 Department of Research and Development, Oslo Metropolitan University, Oslo, Norway; 18 Department of Oral Biology, Faculty of Dentistry, University of Oslo, Oslo, Norway; 19 National Centre for Optics, Vision and Eye Care, Department of Optometry, Radiography and Lighting Design, Faculty of Health Sciences, University of South-Eastern Norway, Kongsberg, Norway; 20 Department of Health and Nursing Science, The Faculty of Health and Sport Sciences, University of Agder, Grimstad, Norway; 21 Department of Quality and Health Technology, The Faculty of Health Sciences, University of Stavanger, Stavanger, Norway

**Keywords:** Eye diseases, Computer science, Medical research

## Abstract

Dry eye disease is one of the most common ophthalmological complaints and is defined by a loss of tear film homeostasis. Establishing a diagnosis can be time-consuming, resource demanding and unpleasant for the patient. In this pilot study, we retrospectively included clinical data from 431 patients with dry eye disease examined in the Norwegian Dry Eye Clinic to evaluate how artificial intelligence algorithms perform on clinical data related to dry eye disease. The data was processed and subjected to numerous machine learning classification algorithms with the aim to predict decreased tear film break-up time. Moreover, feature selection techniques (information gain and information gain ratio) were applied to determine which clinical factors contribute most to an unstable tear film. The applied machine learning algorithms outperformed baseline classifications performed with *ZeroR* according to included evaluation metrics. Clinical features such as ocular surface staining, meibomian gland expressibility and dropout, blink frequency, osmolarity, meibum quality and symptom score were recognized as important predictors for tear film instability. We identify and discuss potential limitations and pitfalls.

## Introduction

The ocular tear film consists of an inner mucoaqueous layer and an outer lipid layer^1^. The outer lipid layer is hypothesized to be comprised of an inner amphipathic lipid layer and an outer nonpolar lipid layer^2^. The tear film lipid layer acts as a hydrophobic barrier that reduces evaporation of the underlying aqueous component, protects the eye from damage by external agents and lubricates the ocular surface^1^. The bulk of the tear film lipid layer is produced by modified sebaceous glands located in the upper and lower eyelids referred to as meibomian glands (MG)^3^.

Dry eye disease (DED) is defined as “a multifactorial disease of the ocular surface characterized by a loss of homeostasis of the tear film, and accompanied by ocular symptoms, in which tear film instability and hyperosmolarity, ocular surface inflammation and damage, and neurosensory abnormalities play etiological roles”^4^. It is a multifactorial disease caused by loss of tear film homeostasis that affects 5–50% of the population, depending on the definition and population studied^4,5^. The two main aetiologies are aqueous-deficient dry eye (ADDE) and evaporative dry eye (EDE)^4^. ADDE, caused by lacrimal failure, is often divided into Sjögren and non-Sjögren lacrimal disease and accounts for approximately 10% of the DED incidence^6^. Evaporative dry eye disease is about nine times more common than ADDE, and meibomian gland dysfunction (MGD) is the most common cause^4^.

The diagnostic loop is often initiated with symptom screening through questionnaires such as the Dry Eye Questionnaire 5 (DEQ5) or the Ocular Surface Disease Index (OSDI), followed by clinical tests evaluating the quantity and quality of the tear film^7^. Common clinical tests include the Schirmer test (ST), which quantifies tear production over 5 min, tear film break-up time (TBUT), ocular surface staining (OSS), MG expressibility (ME), meibum quality (MQ) and osmolarity. Further diagnostic work-up may include imaging techniques evaluating quality and quantity of the MGs. Meibography through non-contact infrared imaging has gained clinical and scientific momentum over the past decade^8^.

Artificial intelligence (AI) has been defined as “the science and engineering of making intelligent machines”^9^. Machine learning (ML) is a subgroup of AI focused on the programming of computers enabling them to learn from data through experience and performance improvement over time without being explicitly programmed^10,11^. ML algorithms learn from data through a process referred to as training. The resultant program is a ML model capable of making predictions on data similar to the data it was trained on. ML training methods are categorized based on the type of data used/available; the three main groups include supervised learning, unsupervised learning, and reinforcement learning. In supervised learning the model is trained on data in which the target value is known, referred to as labelled data. Typical supervised learning tasks include classification and regression. Following training, performance is evaluated by letting the model predict on a separate test set and comparing the predictions of the model to the actual labels. In unsupervised learning, models are constructed through training on unlabelled data and are mostly used for clustering and dimensionality reduction. Model performance can be based on e.g. human level performance or distance between cluster points, as the actual labels are unknown^12,13^. Reinforcement learning, on the other hand, is based on continuous feedback to the learning system in form of penalties and/or rewards based on the actions of the learner^10^. The system learns the best possible actions in each state of the system to get as much accumulated rewards as possible. Deep learning is another subfield of ML based on artificial neural networks with more than one hidden layer (deep neural networks). These neural networks are loosely based on their biological counterparts and their architecture typically consist of an input layer, several hidden layers and an output layer. Unlike most traditional ML algorithms, deep learning does not require extensive manual feature engineering. Both supervised and unsupervised approaches may be employed, and deep learning is often used for text and image recognition.

ML has gained great momentum in the field of medicine and ophthalmology^14^. In its application in DED, ML has largely been used for image analysis from slit lamp examinations^15^, meibography^16^, and in vivo confocal microscopy^17^, as well as a few studies concerning osmolarity^18^, and proteomic analysis^19^. Additionally, our group recently employed dimensionality reduction through principal components analysis to compare salivary and ocular lipids and lipidomic profiles in patients with Sjögren`s syndrome to healthy controls^20^. There is no definitive test for DED, and patients must undergo several and occasionally unpleasant clinical examinations demanding a great deal of time and resources. Identifying superfluous clinical tests whose results can be predicted based on other findings might help to reduce the number of tests necessary, minimizing the impact of the examination upon the patient. With this in mind, tabulated clinical data from the Norwegian Dry Eye Clinic was analysed through supervised learning. The present project was undertaken to evaluate if well-known ML algorithms are capable of classifying patients according to TBUT based on tabulated clinical data in DED. Moreover, the project aimed to assess whether new important features might be identified, solidify known associations, and to identify limitations and pitfalls. Prior work has estimated TBUT from video frames using ML^21–23^. There is to our knowledge no studies implementing ML algorithms on tabular clinical data of DED patients.

## Methods

### Participants

Clinical data from 432 patients was extracted from a comprehensive dataset collected between 2017 and 2019 in the Norwegian Dry Eye Clinic in this retrospective study. The clinical data was transformed into datasets for use in this and future projects. Inclusion criteria were adult patients visiting the Norwegian Dry Eye Clinic within the project period, having a diagnosis of DED, and ability to provide informed consent. No specific exclusion criteria were formulated for inclusion of clinical data in the present project.

Written informed consent was obtained from all subjects and the study was approved by the Regional Medical Ethics Committee of South-East Norway (2017/389; approval date 18 April 2017), all performed procedures were in compliance with the Declaration of Helsinki.

### Dry eye examinations

All clinical examinations were performed as previously described by experienced ophthalmologists specialised in DED^24^. In brief, following completion of the OSDI questionnaire, ocular surface tests were performed in the order: tear film osmolarity, TBUT (measured with fluorescein under slit lamp microscopy, average of three measurements used), OSS (assessed according to the Oxford grading scheme^25^), corneal sensitivity (Cochet-Bonnet esthesiometer), ST (without anesthesia), ME and MQ. Blink frequency was counted prior to examinations while patients were conducting the OSDI questionnaire. Patients were diagnosed with DED if the OSDI was ≥ 13 and/or either TBUT < 10 s or OSS > 0. Meibographic images were acquired through the Keratograph 5 M (Oculus Optikgeräte GmbH, Wetzlar, Germany). Degree of meibomian gland dropout was graded subjectively according to the meibograde by Pult et al.^26^. Images were taken of both eyelids on both eyes if possible and results averaged to a composite score for each eye.

### Data preparation

Patient characteristics, including demographic, clinical, and proteomic data were initially recorded in an Excel (Microsoft Corporation, 2018. Microsoft Excel, Available at: https://office.microsoft.com/excel) spreadsheet. A thorough review of the spreadsheet was performed, and relevant features selected. Features were converted to uniform numerical data of similar grading systems where necessary (e.g. to meiboscale by Pult et al.^26^, rather than percentage dropout) to ensure data homogeneity for a given feature, and blank cells reformatted to “999”. The .xlsx file was imported and converted to the .arff file format through Weka (Waikato Environment for Knowledge Analysis, University of Waikato, New Zealand)^27^. Clinical features included in the final analysis were age, age group 0–19, age group 20–39, age group 40–59, age group 60–79, age group 80–99, sex, OSDI, ST OD (right eye), ST OS (left eye), osmolarity OD, osmolarity OS, meibography OD, meibography OS, ME OD, ME OS, MQ OD, MQ OS, blink frequency OD, blink frequency OS, corneal sensibility OD, corneal sensibility OS, OSS OD, OSS OS, and the class to be predicted (TBUT OD or TBUT OS). TBUT was measured in whole seconds, < 10 s was considered pathological. The TBUT class was divided into three groups, with group 1 (ranging 0–4 s), group 2 (5–9 s) and group 3 (≥ 10 s). TBUT values as a running numerical class was removed as a feature for both eyes to avoid information leakage and consequential false overperformance of the ML models.

### Analysis

The final dataset consisted of 431 instances and 25 features; it is not public available as it contains sensitive patient data.

Analysis was performed in the Weka library version 3.8.6 (Weka 3—Data Mining with Open Source Machine Learning Software in Java (waikato.ac.nz)) with TBUT at baseline in both eyes as the classes to be predicted. *ZeroR* was run as the baseline classifier. *ZeroR* is considered one of the simplest rule-based classifiers, it ignores all predictors and relies on the target^28^. Based on a frequency table it predicts the majority class. It predicts the mode for nominal data and the mean for numeric data. As a classifier it is considered difficult to beat with respect to certain metrics, such as accuracy, if the data is biased, due to classification of all instances to the majority class.

After calculating the *ZeroR* classification baseline, the performance of other algorithms were evaluated. Algorithms were chosen based on empirical testing (in total we tested 40 different algorithms). The algorithms presented in this work are the best known and highest performing algorithms tested. Detailed information regarding classifier evaluation and results were saved for comparison. The top classifiers that are also reported in this work are random forest, multilayer perceptron, *AdaBoostM1*, *LogitBoost* and randomizable filtered classifier. All hyperparameters of the classifiers were set to the optimal standard settings provided by Weka.

Decision trees predict the value of a target feature based on input features and can perform both regression and classification tasks^10^. A random forest is made up of an assortment of decision trees. Upon performing classification tasks, the output of the random forest is the class predicted by the highest number of decision trees within the forest. The random forest algorithm trades a higher bias for a lower variance through greater diversity by searching for the best feature among a random subset of features upon splitting a node, resulting in an overall superior model.

For the sake of including models of various categories, we have included the results from classification with naïve Bayes. This algorithm is a simple probabilistic classifier based on Bayes` theorem^29^. It is based on the probability of observing predictor values given an outcome, to calculate the probability of an outcome based on the feature values.

The multilayer perceptron is an artificial neural network consisting of an input layer, at least one hidden layer and an output layer. The multilayer perceptron employs a technique referred to as backpropagation to calculate the error rate of the model and tweak the input of weights and bias to reduce the overall error.

*AdaBoost* (Adaptive Boosting) boosts nominal class classifiers by running a “weak” learning algorithm over numerous allotments of the training data and then combining these into a single classifier^30^. The main weakness of this model stems from the random guessing of the label for a hypothesis with expected error 1–1/k where k is the number of possible labels. Hence, when k > 2, there is an increased requirement that the error be < ½.

*LogitBoost*, like *AdaBoost*, performs additive logistic regression^31^. However, whereas adaptive boosting minimizes the exponential loss, logistic boosting minimizes the logistic loss.

The randomizable filtered classifier is a version of the filtered classifier that concretize the model with a randomizable filter. It implements *IBk*, a K-nearest neighbour’s classifier, as the base classifier.

Based on the results obtained when predicting the TBUT of the right eye at baseline, the best performing classifiers were also employed to predict the OS class.

Determination of which features had the greatest impact on the classification, and to what degree, was analysed using algorithms that can determine the feature importance for a given classification task. Specifically, we used the information gain and information gain ratio algorithms. This was done to evaluate what clinical features being most important in producing an unstable tear film.

### Metrics

To evaluate the performance of the algorithms we used a set of metrics to get a better understanding. The metrics were chosen based on insights obtained from^32^. Specifically, we used recall, false positive rate, precision, F-measure, and Matthews correlation coefficient (MCC). Included in these metrics are true positive (TP), true negative (TN), false positive (FP) and false negative (FN).

Recall, also known as sensitivity or true positive (TP) rate, is the fraction of correctly classified positive instances. It is bounded to [0, 1] where 1 represents perfect prediction of the positive class and 0 incorrect prediction of all samples.

1 $$Recall=\frac{True\, positives}{Total \,number \,of\, positives}=\frac{TP}{TP+FN}.$$

FP rate reflects the probability of falsely rejecting the null hypothesis. The FP rate is bounded [0, 1], where 0 represents no false positives predicted and 1 that all cases are wrongly predicted as positive.

2 $$FP\, rate=\frac{FP}{FP+TN}.$$

Precision, also known as positive predictive value, is the ratio of TP and the total number predicted as positive by a model. It is bounded [0, 1] where 0 represents no correct predictions and 1 represents all predictions correct.

3 $$Precision=\frac{Correctly \,classified \,samples}{Samples \,assigned\, to\, class}=\frac{TP}{TP+FP}.$$

The F-measure, or F1-score, is the harmonic mean of precision and recall. It penalizes extreme values of both and is used to evaluate the accuracy of predictions. It is bounded [0, 1] where 1 represents perfect precision and recall and 0 represents no precision and recall.

4 $$F1\, score=2*\frac{precision*recall}{precision+recall}=\frac{2*TP}{2*TP+FP+FN}.$$

MCC is a correlation coefficient between true and predicted classes. It is bounded [− 1, 1] where − 1 represents total disagreement between the true value and prediction, 0 equals random guessing and 1 represents perfect prediction. As it includes all entries from the confusion matrix a high value necessitates globally good results.

5 $$MCC=\frac{TP*TN-FP*FN}{\sqrt{\left(TP+FP\right)\left(TP+FN\right)\left(TN+FP\right)\left(TN+FN\right)}}.$$

References for Eqs. (1–5) can be found in^32,33^. We split the data into 50% training and 50% test data. On the training data we performed tenfold cross validation, a method where the training set is randomly divided into ten subsets (folds)^10^. The model is trained and evaluated 10 times using a different subset of the data for validation every time while training on the remaining nine folds. The resulting model was tested on the 50% test data split. Hyperparameter optimization was performed using GridSearch on the best performing algorithms. This did not enhance performance on any model with the exception of naïve Bayes. For future work we will test the model trained on the full dataset in a prospective study to validate the clinical applicability of the best methods identified in this work.

## Results

The patients were from 18 to 88 years old, with a mean age of 52.7 years. 101 patients were male and 330 were female. One patient was excluded from the dataset and analysis as the age was registered as “0”. Demographic and clinical data is presented in Table 1. All results are available in the supplementary materials (Supplementary File 1).

**Table 1:** Demographics and clinical data.

Sex	Frequency			
Men	101			
Women	330			
Parameter	Min	Max	Mean	SD
Age	18	88	52.65	21.21
**Schirmer test (mm/5 min)**				
Right eye	0	35	12.61	8.85
Left eye	0	36	13.98	8.88
**TBUT (seconds)**				
Right eye	1	14	3.45	2.67
Left eye	1	16	3.54	2.96
**OSS**				
Right eye	0	9	1.83	1.75
Left eye	0	8	2.12	2.02
**ME**				
Right eye	0	3	2.17	0.86
Left eye	0	3	2.25	0.84
**MQ**				
Right eye	0	24	9.86	5.71
Left eye	0	22	10.27	5.90
**Blink frequency (blinks/minute)**				
Right eye	12	52	26.52	8.43
Left eye	12	52	26.52	8.43
**Corneal sensibility**				
Right eye	30	60	57.42	5.54
Left eye	15	60	57.30	7.35
**Osmolarity (mOsm/L)**				
Right eye	281	313	296.45	9.30
Left eye	276	329	296.07	13.41
**Meibography (meiboscale by Pult et al.)**				
Right eye	0	4	2.31	1.14
Left eye	0	4	2.34	1.12
OSDI	0	80	28.62	18.83

*Max* maximum, *ME* meibomian gland expressibility, *MQ* meibum quality, *Min* minimum, *mm* millimetres, *mOsm/L* milliosmole/litre, *OSDI* ocular surface disease index, *OSS* ocular surface staining, *SD* standard deviation, *TBUT* tear film break up time.

### Right eye at baseline

In the right eye groups 1, 2 and 3 had 334, 61 and 24 instances respectively. Moreover, 12 instances had blank values and was assigned to group “999”. Results are shown in Table 2.

**Table 2:** Supervised learning results on the right eye.

Scheme	Recall	FP rate	Precision	F-measure	MCC	Correctly classified
ZeroR class 1	1.0	1.0	0.775	0.873	NC	
ZeroR class 2	0.0	0.0	NC	NC	NC	
ZeroR class 3	0.0	0.0	NC	NC	NC	
ZeroR weighted avg	0.775	0.775	NC	NC	NC	77.494%
NaiveBayes class 1	1.0	0.522	0.876	0.934	0.650	
NaiveBayes class 2	0.469	0.0	1.0	0.638	0.660	
NaiveBayes class 3	0.417	0.0	1.0	0.588	0.640	
NaiveBayes weighted avg	0.888	0.410	0.902	0.871	0.650	88.837%
Random forest class 1	1.0	0.010	0.997	0.999	0.993	
Random forest class 2	0.984	0.0	1.0	0.992	0.990	
Random forest class 3	1.0	0.0	1.0	1.0	1.0	
Random forest class weighted avg	0.998	0.008	0.998	0.998	0.993	99.768%
Multilayer perceptron class 1	0.994	0.072	0.979	0.987	0.940	
Multilayer perceptron class 2	0.967	0.005	0.967	0.967	0.962	
Multilayer perceptron class 3	0.792	0.0	1.0	0.884	0.884	
Multilayer perceptron weighted avg	0.979	0.057	0.979	0.979	0.941	97.912%
Randomizable filtered classifier + IBk class 1	1.0	0.0	1.0	1.0	1.0	
Randomizable filtered classifier + IBk class 2	1.0	0.0	1.0	1.0	1.0	
Randomizable filtered classifier + IBk class 3	1.0	0.0	1.0	1.0	1.0	
Randomizable filtered classifier + IBk weighted avg	1.0	0.0	1.0	1.0	1.0	100%

*FP* false positive, *MCC* Matthews correlation coefficient, *avg*. average, *NC* not calculable.

Classification through *ZeroR* gave 77.494% correctly classified instances. For class 1 it demonstrated a recall and FP rate of 1.0, precision of 0.775 and a F-measure of 0.873. MCC could not be calculated because only majority class labels are assigned.

Using *RandomForest* with 100 iterations correctly classified instances increased to 99.768%. The weighted average for recall was 0.998 and the FP rate was 0.008. Precision, F-measure and MCC were 0.998, 0.998 and 0.993, respectively. As opposed to *ZeroR*, the confusion matrix after *RandomForest* demonstrated improvement with increased diagonal dispersion, representing more correctly classified instances.

*MultilayerPerceptron* with “t” hidden layers (t = features + classes) and 500 epochs gave 97.912% correctly classified instances.

*AdaBoostM1* and *LogitBoost* with *RandomForest* as the sub classifier gave exactly the same results on all metrics as *RandomForest* alone, with only one wrong prediction.

The randomizable filtered classifier with *IBk* as the base classifier gave 100% correctly classified instances with recall = 1.0, FP = 0.0, Precision = 1.0, F-measure = 1.0 and MCC = 1.0 in weighted average.

### Left eye at baseline

In the left eye groups 1, 2 and 3 had 323, 60 and 36 instances respectively. Also, 12 instances had blank values and was assigned to group “999”. Results are shown in Table 3.

**Table 3:** Supervised learning results on the left eye.

Scheme	Recall	FP rate	Precision	F-measure	MCC	Correctly classified
ZeroR class 1	1.0	1.0	0.749	0.857	NC	
ZeroR class 2	0.0	0.0	NC	NC	NC	
ZeroR class 3	0.0	0.0	NC	NC	NC	
ZeroR weighted avg	0.749	0.749	NC	NC	NC	74.942%
NaiveBayes class 1	1.0	0.964	0.746	0.855	0.163	
NaiveBayes class 2	0.0	0.0	NC	NC	NC	
NaiveBayes class 3	0.0	0.0	NC	NC	NC	
NaiveBayes weighted avg	0.749	0.713	NC	NC	NC	74.884%
Random forest class 1	1.0	0.0	1.0	1.0	1.0	
Random forest class 2	1.0	0.0	1.0	1.0	1.0	
Random forest class 3	1.0	0.0	1.0	1.0	1.0	
Random forest weighted avg	1.0	0.0	1.0	1.0	1.0	100%
Randomizable filtered classifier + IBk class 1	0.994	0.0	1.0	0.997	0.988	
Randomizable filtered classifier + IBk class 2	1.0	0.0	1.0	1.0	1.0	
Randomizable filtered classifier + IBk class 3	1.0	0.005	0.947	0.973	0.971	
Randomizable filtered classifier + IBk weighted avg	0.995	0.0	0.996	0.995	0.988	99.536%

*FP* false positive, *MCC* Matthews correlation coefficient, *avg*. average, *NC* not calculable.

*ZeroR* classification demonstrated 74.942% correctly classified instances with a recall and FP rate of 1.0, Precision = 0.749 and F-measure = 0.857 for class 1. As with OD, the MCC could not be calculated.

*RandomForest* gave 100% correctly classified instances with recall = 1.0, FP = 0.0, Precision = 1.0, F-measure = 1.0 and MCC = 1.0 in weighted average.

*LogitBoost* and *AdaBoostM1* with *RandomForest* as the sub classifier gave exactly the same results as *RandomForest* alone. Perfect predictions were also achieved through *IBk* and *KStar* (K*, instance-based K-nearest neighbour classifier).

Only minor differences in the predictive capabilities of the included algorithms for OD and OS can be found, with generally slightly higher percentage of correctly classified instances seen in OD. *NaiveBayes* had 14% more correct instances in OD. However, random forest made perfect predictions on OS but not OD. Boosting with either *AdaBoost* or *LogitBoost* with random forest as the subclassifier made no difference on the results in either OD or OS. The randomizable filtered classifier with *IBk* as the subclassifier made the best predictions on OD with marginally weaker results on OS. The predictive algorithms overcame class imbalance and correctly classified instances to the minority classes 2 and 3, even though the data was skewed.

### Impact of various features

#### Most important features in the right eye

As entropy-based ranking methods are computationally cheap and reliable, results from information gain and information gain ratio are presented^34^. Following analysis, medical implications of the identified features were assessed, this is discussed below.

The information gain-based feature evaluation determines the worth of a feature by measuring the information gain with respect to the class^35^, the ten most important features are listed in Table 4. The values presented for a given feature reflects its contribution to reduce the entropy, calculated by *InfoGain* (Class, Feature) = H (Class) – H (Class | Feature), where a higher score reflects a greater contribution.

**Table 4:** Most important features predicting an unstable tear film in the right eye with information gain.

InfoGainAttributeEval (Information gain)	
Age	0.864
ST, OS	0.763
OSDI	0.480
ST, OD	0.462
MQ, OD	0.348
MQ, OS	0.321
OSS, OD	0.289
OSS, OS	0.286
Blinkfreq., OS	0.266
Blinkfreq., OD	0.266

*OD* right eye, *OS* left eye, *ST* Schirmer test, *MQ* meibum quality, *OSDI* ocular surface disease index, *Blinkfreq* blink frequency, *OSS* ocular surface staining.

The information gain ratio-based evaluation determines the worth of a feature by measuring the gain ratio with respect to the class, the ten most important features are listed in Table 5.

**Table 5:** Most important features predicting an unstable tear film in the right eye with information gain ratio.

GainRatioAttributeEval (Information gain ratio)	
Age	0.264
OSS, OD	0.225
ST, OS	0.218
OSS, OS	0.180
OSDI	0.175
Osm, OS	0.170
ST, OD	0.162
ME, OS	0.161
MQ, OS	0.158
ME, OD	0.150

*OD* right eye, *OS* left eye, *ST* Schirmer test, *ME* meibomian gland expressibility, *MQ* meibum quality, *OSDI* ocular surface disease index, *OSS* ocular surface staining, *Osm* osmolarity.

#### Most important features in the left eye

The ten most important information gain features for the left eye are listed in Table 6.

**Table 6:** Most important features predicting an unstable tear film in the left eye with information gain.

InfoGainAttributeEval (Information gain)	
Age	1.050
ST, OS	0.808
ST, OD	0.747
OSDI	0.511
Blinkfreq., OD	0.423
Blinkfreq., OS	0.423
MQ, OS	0.380
MQ, OD	0.364
OSS, OD	0.360
Osm, OS	0.264

*OD* right eye, *OS* left eye, *ST* Schirmer test, *MQ* meibum quality, *OSDI* ocular surface disease index, *Blinkfreq* blink frequency, *OSS* ocular surface staining, *Osm* osmolarity.

The ten most important information gain ratio features for the left eye are listed in Table 7.

**Table 7:** Most important features predicting an unstable tear film in the left eye with information gain ratio.

GainRatioAttributeEval (Information gain ratio)	
Age	0.288
Meibography, OS	0.264
Meibography, OD	0.264
ST, OS	0.226
OSS, OS	0.216
ST, OD	0.191
Osm, OS	0.190
OSDI	0.183
ME, OS	0.169
Osm, OD	0.167

*OD* right eye, OS left eye, *ST* Schirmer test, *ME* meibomian gland expressibility, *OSDI* ocular surface disease index, *OSS* ocular surface staining, *Osm* osmolarity, *Meibography* degree of MG dropout.

Information gain revealed quite similar features of importance for OD and OS, with the exception of OSS OS being replaced by osmolarity OS when predicting on the left eye. When predicting on the right eye, information gain rates MQ as more important than blink frequency, conversely, this is opposite for the left eye. The dissimilarities are greater concerning important features according to information gain ratio. OSS OD, MQ OS and ME OD are included for the right eye, but not for the left eye. However, in the left eye osmolarity OD and degree of MG dropout for both eyes as determined through meibography are important contributing factors. When comparing results between information gain and information gain ratio age, Schirmer test, OSS, MQ, MG expressibility and dropout, osmolarity, blink frequency and OSDI are the most important features for predicting an unstable tear film in both eyes using both evaluation methods. According to information gain, blink frequency is an important feature in both eyes, but not according to information gain ratio.

## Discussion

The main aim of the present study was to evaluate whether common ML algorithms can make predictions on clinical data in DED. In the right eye, the *ZeroR baseline* resulted in a total of 77.49% correctly classified instances. Furthermore, for class 1, the recall and FP rate was 1.0, with precision of 0.78 and F-measure 0.87. MCC could not be calculated. Excluding the latter and the FP rate, these results are considered difficult to beat. These seemingly strong baseline metrics are a result of bias due to class imbalance as 334/431 instances belong to class 1. In Weka there are ways of counteracting class imbalance problems such as systematic oversampling (SMOTE) and random under sampling. In the former, the software produces synthetic instances for the minority class (es) based on a given number of nearest neighbours. Conversely, in the latter instances in the majority class is randomly removed. Class balancing through SMOTE is often employed to balance skewed datasets. We did not do this due to the excellent predictive capabilities of the algorithms on our unmanipulated dataset. However, as class imbalance is a potential pitfall and source of bias when employing ML algorithms, it should be kept in mind during data collection, preparation and calculation.

Random forest correctly classified 99.77% on the right eye with recall, precision, F-measure and MCC all > 0.99 for class 1. As can be seen in Table 2, these metrics do not deteriorate when predicting on class 2 and 3. Moreover, the FP rate for class 1 is 0.01, indicating that very few instances belonging to class 2 and 3 are wrongly assigned to class 1.

By comparison the multilayer perceptron correctly predicted 97.91% of instances. For class 1 the recall was lower. However, the FP was also slightly decreased, indicating less of a tendency for wrongly assigning instances belonging to class 2 and 3 to class 1. Despite this, the MCC of the multilayer perceptron was lower than that of the random forest with 0.941 vs. 0.993 weighted average, indicating overall poorer classifier performance.

**Figure 1:**
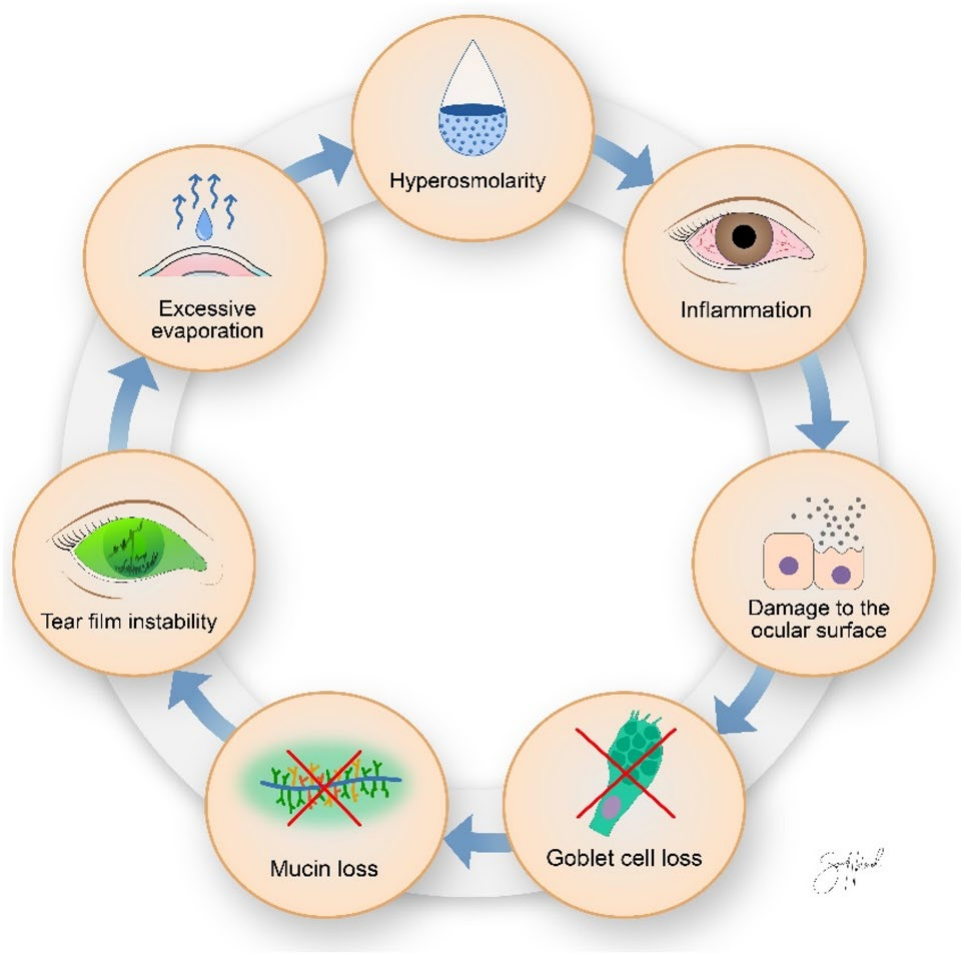
The vicious cycle of dry eye disease. Illustration by Sara Nøland.

Age, OSS, ST and OSDI stand out as the most important predictors upon examining the features associated with an unstable tear film, followed by meibomian gland dropout, expressibility, blink frequency, osmolarity and meibum quality. These findings are in accordance with those described by the Tear Film and Ocular Surface (TFOS) Pathophysiology subcommittee and the vicious cycle of DED as depicted in Fig. 1^36^. This cycle may be entered at any point and propagated by numerous interrelated processes collectively advancing DED. Tear film instability may cause increased evaporation of the underlying watery component with resultant hyperosmolarity, a clinical sign included in the “TFOS Dry Eye Workshop 2” definition of DED^4^. In our findings, changes in osmolarity were considered among the ten most important features in three of the four analyses, corroborating its importance as a pathophysiologic entity of DED. Arita et al. proposed several diagnostic criteria for obstructive MGD based on the correlations of clinical findings in patients compared to healthy controls^37^. They found that ocular symptom score, degree of lid margin abnormalities, MG expressibility and meibum quality, ocular surface staining and degree of MG dropout were significantly higher in patients with MGD.

Past studies have demonstrated correlation between MG dropout, TBUT^38,39^ and OSDI^40^. However, in other studies this correlation is either very weak or absent^41,42^. In our dataset, MG dropout as a feature was ranked as the 2nd and 3rd most important features when predicting decreased TBUT in the left eye, but not in the right using information gain ratio with Ranker.

Our findings that MG expressibility and meibum quality were of importance in predicting decreased tear film stability corroborates the importance of meibum lipids in stabilizing the tear film and preventing evaporation of the underlying aqueous component^1^. As ocular surface staining is a hallmark of longstanding inflammatory changes in DED resulting from damage to the ocular surface, its role as one of the most important predictors for decreased TBUT is understandable^7^. However, whether a decreased TBUT causes ocular surface staining, inflammation destabilizes the tear film, or if it is a mutually degrading relationship, remains unknown. Based on the rationale of the vicious cycle of DED, the latter is an alluring concept.

Blink frequency in the healthy population ranges from 10 to 15 blinks per minute and is increased in DED^7^. A stable tear film that does not dissociate during the interblink interval is vital in protecting the ocular surface. Longer periods between the break-up of the tear film and the subsequent blink, increase the stress inflicted on the ocular surface^43^. Thus, our finding that increased blink frequency is associated with an unstable tear film substantiates this relationship.

Our analyses revealed ST as a significant factor in predicting tear film instability concerning values from both eyes, on both eyes with both algorithms. Hence, it is in our case one of the most consequential predictors. As ST is a measure of tear fluid volume produced over a given period, this relationship might seem counterintuitive. Mathers et al. identified a subgroup of MGD patients with hyperosmolarity, high degree of MG dropout and decreased ST values^44^. This cohort corresponds to the subgroup predicted by Bron et al. where advanced EDE patients suffering from corneal neuropathy loose lacrimal compensation developing a functional ADDE^45^. Indeed, a correlation between decreased ST values, TBUT and lipid layer thickness has been reported^46^. Moreover, a recent study demonstrated that MGD patients with lower ST values had decreased tear film stability compared to healthy controls (not age nor sex matched) and MGD patients with normal ST values^47^.

There are several limitations to the present study. The first and foremost limitation is the retrospective design and that all patients were included on the sole basis of being diagnosed with DED. The lack of exclusion criteria might have resulted in a heterogenous sample with increased prevalence of comorbidities resulting in selection bias. This inclusion model is explained by our aim to examine whether ML algorithms could be used on clinical data in DED and the need for a large number of instances. Another limitation is class imbalance. However, the skewedness of our dataset did not appear to impact the ability of our models to make accurate predictions of the minority classes and maintained a low FP rate in the majority class. Thus, we did not counteract this through synthetic oversampling. Missing values in the dataset were ascribed the value “999”. It is unlikely that this influenced the results as the value is far higher than the range of any included tests and the algorithms are more likely to learn that these are non-relevant outliers. In total 1319/12930 (10.2%) values are missing which is a small percentage of the full dataset and should not have a big effect on the results (especially for methods that can handle missing values). Nevertheless, for future work we would also like to test different imputation methods and their influence on the overall performance. Despite good predictive capabilities, caution is warranted upon drawing conclusions concerning the impact of the different features in affecting the tear film stability. There might be an element of multicollinearity among the included features. Although this will not influence the predictive capabilities of the models as a whole, it might affect the validity in explaining which features are the most important, and this might be the reason for some discrepancy in our results regarding which is the most influential features on TBUT between the right and the left eye.

A strength of the study is the large number of patients, all derived from one site, which reduces the variation in methodological approaches (clinicians and equipment), which otherwise may preclude the data set. However, this might also serve as a limitation as there can be considerable inter-rater variability concerning the various dry eye tests. Thus, it is unknown how well these algorithms would work on data collected from several clinics and clinicians. Moreover, even though a large number of patients is included, this is a relatively low number for training a robust multilayer perceptron and other ML algorithms. This, in combination with a skewed dataset, might have caused overfitting of models influencing the results. The relatively small sample size and skewedness of the dataset are also the reasons why tenfold cross validation was used on the training data split in order to obtain a more robust model and prevent overfitting the algorithms. Hyperparameter optimization was performed with *GridSearch*. This only improved naïve Bayes, possibly due to overfitting. The best performing algorithms of this study will be further evaluated in a prospective study. Another strength is the large number of algorithms employed, several of which made accurate predictions, substantiating the role of ML models in future works.

Despite the limitations of the current study, we conclude that ML algorithms are capable of making accurate predictions on TBUT based on tabular clinical data in DED. Further studies are warranted to examine whether other clinical outcomes might be successfully predicted based on tabular data. If these findings are confirmed in larger, prospective studies, preferably on balanced datasets, they might indicate clinical examinations that are superfluous and, thus, might be omitted from the standard work-up. If this is achievable, it will reduce patient discomfort as well save time and resources for clinicians. In addition to demonstrating that accurate predictions can be made on tabular clinical data in DED, herein exemplified using TBUT, the present work helps to substantiate and solidify known associations between several clinical features, such as age, OSS, ST, OSDI and their effect on tear film stability. Our study provides new and important information as all diagnostics have the goal to ensure optimal therapy. In our study, we have taken an innovative AI based approach to reveal the most important factors associated with low and high TBUT. Based on this understanding, the therapy can more easily be optimized. For example, a clear association with MQ and TBUT, should bring the therapeutic focus to the clinicians to what is scientifically well-documented to improve MQ. This will vary over time but can include strategies such as intense pulsed light therapy^48^. TBUT is chosen in our study as it is a critical diagnostic parameter in all DED management. A major strength of the methodology described in this article is the versatility of ML algorithms. Once a dataset is collected one can relatively easily change what feature to predict. As we herein have demonstrated the ability of these algorithms to make accurate predictions on DED clinical data, this not only enables researchers to pinpoint clinical features easily predicted, but also through which examinations this might be done. An additional possibility is the combination of clinical data, ML algorithms and proteomic and/or lipidomic measurements. As these biochemical analyses produce large amounts of data, they are ideal candidates for predictions and clustering through both supervised and unsupervised learning algorithms. We believe these methodologies will help solidify the importance of established features and that novel connections may be identified. Identification of biochemical profiles typical of DED subgroups, how these subgroups respond to various treatment modalities and how to predict which treatment will be the most beneficial for a given patient might be possible. Studies with larger, balanced datasets, with a higher number of features are needed and currently underway based on the findings in this pilot study.

## Supplementary Information

Supplementary Information.

## Data Availability

The dataset is not publicly available as it contains sensitive patient data. Access to processed data used for analysis can be given upon request by contacting the corresponding author.
