# Supplementary material for: Predicting an unstable tear film through artificial intelligence
Source: Sci Rep. 2022 Dec 10;12:21416. doi: 10.1038/s41598-022-25821-y (PMC9741582; doi:10.1038/s41598-022-25821-y)
Supplement: Supplementary file 1 — Supplementary Information. [file 41598_2022_25821_MOESM1_ESM.pdf]

Predicting an unstable tear film through artificial intelligence

Fredrik Fineide, Andrea Marheim Storås, Xiangjun Chen, Morten S. Magnø, Anis Yazidi, Michael A. Riegler, Tor Paaske Utheim

Analysis OD:

Scheme		Recall	FP Rate	Precision	F-Measure	MCC	Class	Correct	Incorrect								
Scheme:	weka.classifiers.rules.ZeroR	1	1	0.775	0.873	?	1			a	b	c	d	<--	classified	as	
Scheme:	weka.classifiers.rules.ZeroR	0	0	?	?	?	2			##	0	0	0	|	a	=	1
Scheme:	weka.classifiers.rules.ZeroR	0	0	?	?	?	3			61	0	0	0	|	b	=	2
Scheme:	weka.classifiers.rules.ZeroR	0	0	?	?	?	999			24	0	0	0	|	c	=	3
Scheme:	weka.classifiers.rules.ZeroR	0.775	0.775	?	?	?	Weighted ave.	77.49	22.506	12	0	0	0	|	d	=	##
Scheme:	weka.classifiers.rules.DecisionTable -X 1 -S "weka.attributeSelection.BestFirst -D 1 -N 5"	1	0.041	0.988	0.994	1	1			a	b	c	d	<--	classified	as	
Scheme:	weka.classifiers.rules.DecisionTable -X 1 -S "weka.attributeSelection.BestFirst -D 1 -N 5"	0.934	0	1	0.966	1	2			##	0	0	0	|	a	=	1
Scheme:	weka.classifiers.rules.DecisionTable -X 1 -S "weka.attributeSelection.BestFirst -D 1 -N 5"	1	0	1	1	1	3			4	57	0	0	|	b	=	2
Scheme:	weka.classifiers.rules.DecisionTable -X 1 -S "weka.attributeSelection.BestFirst -D 1 -N 5"	1	0	1	1	1	999			0	0	24	0	|	c	=	3
Scheme:	weka.classifiers.rules.DecisionTable -X 1 -S "weka.attributeSelection.BestFirst -D 1 -N 5"	0.991	0.032	0.991	0.991	1	Weighted ave.	99.07	0.9281	0	0	0	#	|	d	=	##
Scheme:	weka.classifiers.rules.JRip -F 3 -N 2.0 -O 2 -S 1	0.997	0.01	0.997	0.997	1	1			a	b	c	d	<--	classified	as	
Scheme:	weka.classifiers.rules.JRip -F 3 -N 2.0 -O 2 -S 1	0.984	0.003	0.984	0.984	1	2			##	1	0	0	|	a	=	1
Scheme:	weka.classifiers.rules.JRip -F 3 -N 2.0 -O 2 -S 1	1	0	1	1	1	3			1	60	0	0	|	b	=	2
Scheme:	weka.classifiers.rules.JRip -F 3 -N 2.0 -O 2 -S 1	1	0	1	1	1	999			0	0	24	0	|	c	=	3
Scheme:	weka.classifiers.rules.JRip -F 3 -N 2.0 -O 2 -S 1	0.995	0.008	0.995	0.995	1	Weighted ave.	99.54	0.464	0	0	0	#	|	d	=	##
Scheme:	weka.classifiers.rules.OneR -B 6	0.985	0.237	0.935	0.959	0.8	1			a	b	c	d	<--	classified	as	
Scheme:	weka.classifiers.rules.OneR -B 6	0.705	0.011	0.915	0.796	0.8	2			##	4	1	0	|	a	=	1
Scheme:	weka.classifiers.rules.OneR -B 6	0.792	0.015	0.76	0.776	0.8	3			13	43	5	0	|	b	=	2
Scheme:	weka.classifiers.rules.OneR -B 6	0.583	0	1	0.737	0.8	999			5	0	19	0	|	c	=	3
Scheme:	weka.classifiers.rules.OneR -B 6	0.923	0.186	0.924	0.92	0.8	Weighted ave.	92.34	7.6566	5	0	0	7	|	d	=	##
Scheme:	weka.classifiers.rules.PART -C 0.25 -M 2	0.991	0.01	0.997	0.994	1	1			a	b	c	d	<--	classified	as	
Scheme:	weka.classifiers.rules.PART -C 0.25 -M 2	0.984	0	1	0.992	1	2			##	0	3	0	|	a	=	1
Scheme:	weka.classifiers.rules.PART -C 0.25 -M 2	1	0.007	0.889	0.941	0.9	3			1	60	0	0	|	b	=	2
Scheme:	weka.classifiers.rules.PART -C 0.25 -M 2	1	0	1	1	1	999			0	0	24	0	|	c	=	3
Scheme:	weka.classifiers.rules.PART -C 0.25 -M 2	0.991	0.008	0.991	0.991	1	Weighted ave.	99.07	0.9281	0	0	0	#	|	d	=	##
Scheme:	weka.classifiers.trees.DecisionStump	1	0.876	0.797	0.887	0.3	1			a	b	c	d	<--	classified	as	
Scheme:	weka.classifiers.trees.DecisionStump	0	0	?	?	?	2			##	0	0	0	|	a	=	1
Scheme:	weka.classifiers.trees.DecisionStump	0	0	?	?	?	3			61	0	0	0	|	b	=	2
Scheme:	weka.classifiers.trees.DecisionStump	1	0	1	1	1	999			24	0	0	0	|	c	=	3
Scheme:	weka.classifiers.trees.DecisionStump	0.803	0.679	?	?	?	Weighted ave.	80.28	19.722	0	0	0	#	|	d	=	##
Scheme:	weka.classifiers.trees.HoeffdingTree -L 2 -S 1 -E 1.0E-7 -H 0.05 -M 0.01 -G 200.0 -N 0.0	1	1	0.775	0.873	?	1			a	b	c	d	<--	classified	as	
Scheme:	weka.classifiers.trees.HoeffdingTree -L 2 -S 1 -E 1.0E-7 -H 0.05 -M 0.01 -G 200.0 -N 0.0	0	0	?	?	?	2			##	0	0	0	|	a	=	1
Scheme:	weka.classifiers.trees.HoeffdingTree -L 2 -S 1 -E 1.0E-7 -H 0.05 -M 0.01 -G 200.0 -N 0.0	0	0	?	?	?	3			61	0	0	0	|	b	=	2
Scheme:	weka.classifiers.trees.HoeffdingTree -L 2 -S 1 -E 1.0E-7 -H 0.05 -M 0.01 -G 200.0 -N 0.0	0	0	?	?	?	999			24	0	0	0	|	c	=	3
Scheme:	weka.classifiers.trees.HoeffdingTree -L 2 -S 1 -E 1.0E-7 -H 0.05 -M 0.01 -G 200.0 -N 0.0	0.775	0.775	?	?	?	Weighted ave.	77.49	22.506	12	0	0	0	|	d	=	##
Scheme:	weka.classifiers.trees.J48 -C 0.25 -M 2	0.985	0.021	0.994	0.989	1	1			a	b	c	d	<--	classified	as	
Scheme:	weka.classifiers.trees.J48 -C 0.25 -M 2	0.967	0.011	0.937	0.952	0.9	2			##	4	1	0	|	a	=	1
Scheme:	weka.classifiers.trees.J48 -C 0.25 -M 2	1	0.002	0.96	0.98	1	3			2	59	0	0	|	b	=	2
Scheme:	weka.classifiers.trees.J48 -C 0.25 -M 2	1	0	1	1	1	999			0	0	24	0	|	c	=	3
Scheme:	weka.classifiers.trees.J48 -C 0.25 -M 2	0.984	0.018	0.984	0.984	1	Weighted ave.	98.38	1.6241	0	0	0	#	|	d	=	##
Scheme:	weka.classifiers.trees.LMT -I -1 -M 15 -W 0.0	0.985	0.052	0.985	0.985	0.9	1			a	b	c	d	<--	classified	as	
Scheme:	weka.classifiers.trees.LMT -I -1 -M 15 -W 0.0	0.984	0.011	0.938	0.96	1	2			##	4	1	0	|	a	=	1
Scheme:	weka.classifiers.trees.LMT -I -1 -M 15 -W 0.0	1	0.01	0.857	0.923	0.9	3			1	60	0	0	|	b	=	2
Scheme:	weka.classifiers.trees.LMT -I -1 -M 15 -W 0.0	0.417	0	1	0.588	0.6	999			0	0	24	0	|	c	=	3
Scheme:	weka.classifiers.trees.LMT -I -1 -M 15 -W 0.0	0.97	0.042	0.972	0.967	0.9	Weighted ave.	96.98	3.0162	4	0	3	5	|	d	=	##
Scheme:	weka.classifiers.trees.RandomTree -K 0 -M 1.0 -V 0.001 -S 1	0.997	0	1	0.999	1	1			a	b	c	d	<--	classified	as	
Scheme:	weka.classifiers.trees.RandomTree -K 0 -M 1.0 -V 0.001 -S 1	1	0.003	0.984	0.992	1	2			##	1	0	0	|	a	=	1
Scheme:	weka.classifiers.trees.RandomTree -K 0 -M 1.0 -V 0.001 -S 1	1	0	1	1	1	3			0	61	0	0	|	b	=	2
Scheme:	weka.classifiers.trees.RandomTree -K 0 -M 1.0 -V 0.001 -S 1	1	0	1	1	1	999			0	0	24	0	|	c	=	3
Scheme:	weka.classifiers.trees.RandomTree -K 0 -M 1.0 -V 0.001 -S 1	0.998	0	0.998	0.998	1	Weighted ave.	99.77	0.232	0	0	0	#	|	d	=	##
Scheme:	weka.classifiers.trees.REPTree -M 2 -V 0.001 -N 3 -S 1 -L -1 -I 0.0	0.991	0.103	0.971	0.981	0.9	1			a	b	c	d	<--	classified	as	
Scheme:	weka.classifiers.trees.REPTree -M 2 -V 0.001 -N 3 -S 1 -L -1 -I 0.0	0.902	0.005	0.965	0.932	0.9	2			##	2	1	0	|	a	=	1
Scheme:	weka.classifiers.trees.REPTree -M 2 -V 0.001 -N 3 -S 1 -L -1 -I 0.0	0.792	0.005	0.905	0.844	0.8	3			5	55	1	0	|	b	=	2
Scheme:	weka.classifiers.trees.REPTree -M 2 -V 0.001 -N 3 -S 1 -L -1 -I 0.0	1	0	1	1	1	999			5	0	19	0	|	c	=	3
Scheme:	weka.classifiers.trees.REPTree -M 2 -V 0.001 -N 3 -S 1 -L -1 -I 0.0	0.968	0.081	0.967	0.967	0.9	Weighted ave.	96.75	3.2483	0	0	0	#	|	d	=	##
Scheme:	weka.classifiers.trees.RandomForest -P 100 -I 100 -num-slots 1 -K 0 -M 1.0 -V 0.001 -S 1	1	0.01	0.997	0.999	1	1			a	b	c	d	<--	classified	as	
Scheme:	weka.classifiers.trees.RandomForest -P 100 -I 100 -num-slots 1 -K 0 -M 1.0 -V 0.001 -S 1	0.984	0	1	0.992	1	2			##	0	0	0	|	a	=	1
Scheme:	weka.classifiers.trees.RandomForest -P 100 -I 100 -num-slots 1 -K 0 -M 1.0 -V 0.001 -S 1	1	0	1	1	1	3			1	60	0	0	|	b	=	2
Scheme:	weka.classifiers.trees.RandomForest -P 100 -I 100 -num-slots 1 -K 0 -M 1.0 -V 0.001 -S 1	1	0	1	1	1	999			0	0	24	0	|	c	=	3
Scheme:	weka.classifiers.trees.RandomForest -P 100 -I 100 -num-slots 1 -K 0 -M 1.0 -V 0.001 -S 1	0.998	0.008	0.998	0.998	1	Weighted ave.	99.77	0.232	0	0	0	#	|	d	=	##
Scheme:	weka.classifiers.bayes.BayesNet -D -Q weka.classifiers.bayes.net.search.local.K2 -- -P 1 -S BAYES -E weka.classifiers.bayes.net.estimate.SimpleEstimator -- -A 0.5	0.991	0.124	0.965	0.978	0.9	1			a	b	c	d	<--	classified	as	
Scheme:	weka.classifiers.bayes.BayesNet -D -Q weka.classifiers.bayes.net.search.local.K2 -- -P 1 -S BAYES -E weka.classifiers.bayes.net.estimate.SimpleEstimator -- -A 0.5	0.803	0.008	0.942	0.867	0.9	2			##	3	0	0	|	a	=	1
Scheme:	weka.classifiers.bayes.BayesNet -D -Q weka.classifiers.bayes.net.search.local.K2 -- -P 1 -S BAYES -E weka.classifiers.bayes.net.estimate.SimpleEstimator -- -A 0.5	1	0	1	1	1	3			12	49	0	0	|	b	=	2
Scheme:	weka.classifiers.bayes.BayesNet -D -Q weka.classifiers.bayes.net.search.local.K2 -- -P 1 -S BAYES -E weka.classifiers.bayes.net.estimate.SimpleEstimator -- -A 0.5	1	0	1	1	1	999			0	0	24	0	|	c	=	3
Scheme:	weka.classifiers.bayes.BayesNet -D -Q weka.classifiers.bayes.net.search.local.K2 -- -P 1 -S BAYES -E weka.classifiers.bayes.net.estimate.SimpleEstimator -- -A 0.5	0.965	0.097	0.965	0.964	0.9	Weighted ave.	96.52	3.4803	0	0	0	#	|	d	=	##
Scheme:	weka.classifiers.bayes.NaiveBayes	0.317	0.031	0.972	0.479	0.3	1			a	b	c	d	<--	classified	as	
Scheme:	weka.classifiers.bayes.NaiveBayes	0.902	0.397	0.272	0.418	0.4	2			##	##	91	0	|	a	=	1
Scheme:	weka.classifiers.bayes.NaiveBayes	0.583	0.231	0.13	0.212	0.2	3			3	55	3	0	|	b	=	2
Scheme:	weka.classifiers.bayes.NaiveBayes	1	0	1	1	1	999			0	10	14	0	|	c	=	3
Scheme:	weka.classifiers.bayes.NaiveBayes	0.434	0.093	0.827	0.47	0.3	Weighted ave.	43.39	56.613	0	0	0	#	|	d	=	##
Scheme:	weka.classifiers.bayes.NaiveBayesMultinomial	0.207	0.031	0.958	0.34	0.2	1			a	b	c	d	<--	classified	as	
Scheme:	weka.classifiers.bayes.NaiveBayesMultinomial	0.902	0.546	0.214	0.346	0.3	2			69	##	54	#	|	a	=	1
Scheme:	weka.classifiers.bayes.NaiveBayesMultinomial	0.583	0.14	0.197	0.295	0.3	3			3	55	3	0	|	b	=	2
Scheme:	weka.classifiers.bayes.NaiveBayesMultinomial	1	0.045	0.387	0.558	0.6	999			0	10	14	0	|	c	=	3
Scheme:	weka.classifiers.bayes.NaiveBayesMultinomial	0.348	0.11	0.795	0.344	0.2	Weighted ave.	34.8	65.197	0	0	0	#	|	d	=	##
Scheme:	weka.classifiers.bayes.NaiveBayesMultinomialText -P 0 -M 3.0 -norm 1.0 -lnorm 2.0 -stopwords-handler weka.core.stopwords.Null -tokenizer "weka.core.tokenizers.WordTokenizer -delimiters \" \\r\\n\\t.,;:\\\'\\\"()?!\"" -stemmer weka.core.stemmers.NullStemmer	1	1	0.775	0.873	?	1			a	b	c	d	<--	classified	as	
Scheme:	weka.classifiers.bayes.NaiveBayesMultinomialText -P 0 -M 3.0 -norm 1.0 -lnorm 2.0 -stopwords-handler weka.core.stopwords.Null -tokenizer "weka.core.tokenizers.WordTokenizer -delimiters \" \\r\\n\\t.,;:\\\'\\\"()?!\"" -stemmer weka.core.stemmers.NullStemmer	0	0	?	?	?	2			##	0	0	0	|	a	=	1
Scheme:	weka.classifiers.bayes.NaiveBayesMultinomialText -P 0 -M 3.0 -norm 1.0 -lnorm 2.0 -stopwords-handler weka.core.stopwords.Null -tokenizer "weka.core.tokenizers.WordTokenizer -delimiters \" \\r\\n\\t.,;:\\\'\\\"()?!\"" -stemmer weka.core.stemmers.NullStemmer	0	0	?	?	?	3			61	0	0	0	|	b	=	2
Scheme:	weka.classifiers.bayes.NaiveBayesMultinomialText -P 0 -M 3.0 -norm 1.0 -lnorm 2.0 -stopwords-handler weka.core.stopwords.Null -tokenizer "weka.core.tokenizers.WordTokenizer -delimiters \" \\r\\n\\t.,;:\\\'\\\"()?!\"" -stemmer weka.core.stemmers.NullStemmer	0	0	?	?	?	999			24	0	0	0	|	c	=	3
Scheme:	weka.classifiers.bayes.NaiveBayesMultinomialText -P 0 -M 3.0 -norm 1.0 -lnorm 2.0 -stopwords-handler weka.core.stopwords.Null -tokenizer "weka.core.tokenizers.WordTokenizer -delimiters \" \\r\\n\\t.,;:\\\'\\\"()?!\"" -stemmer weka.core.stemmers.NullStemmer	0.775	0.775	?	?	?	Weighted ave.	77.49	22.506	12	0	0	0	|	d	=	##
Scheme:	weka.classifiers.bayes.NaiveBayesMultinomialUpdateable	0.207	0.031	0.958	0.34	0.2	1			a	b	c	d	<--	classified	as	
Scheme:	weka.classifiers.bayes.NaiveBayesMultinomialUpdateable	0.902	0.546	0.214	0.346	0.3	2			69	##	54	#	|	a	=	1
Scheme:	weka.classifiers.bayes.NaiveBayesMultinomialUpdateable	0.583	0.14	0.197	0.295	0.3	3			3	55	3	0	|	b	=	2
Scheme:	weka.classifiers.bayes.NaiveBayesMultinomialUpdateable	1	0.045	0.387	0.558	0.6	999			0	10	14	0	|	c	=	3
Scheme:	weka.classifiers.bayes.NaiveBayesMultinomialUpdateable	0.348	0.11	0.795	0.344	0.2	Weighted ave.	34.8	65.197	0	0	0	#	|	d	=	##
Scheme:	weka.classifiers.bayes.NaiveBayesUpdateable	0.317	0.031	0.972	0.479	0.3	1			a	b	c	d	<--	classified	as	
Scheme:	weka.classifiers.bayes.NaiveBayesUpdateable	0.902	0.397	0.272	0.418	0.4	2			##	##	91	0	|	a	=	1
Scheme:	weka.classifiers.bayes.NaiveBayesUpdateable	0.583	0.231	0.13	0.212	0.2	3			3	55	3	0	|	b	=	2
Scheme:	weka.classifiers.bayes.NaiveBayesUpdateable	1	0	1	1	1	999			0	10	14	0	|	c	=	3
Scheme:	weka.classifiers.bayes.NaiveBayesUpdateable	0.434	0.093	0.827	0.47	0.3	Weighted ave.	43.39	56.613	0	0	0	#	|	d	=	##
Scheme:	weka.classifiers.functions.Logistic -R 1.0E-8 -M -1 -num-decimal-places 4	0.997	0.01	0.997	0.997	1	1			a	b	c	d	<--	classified	as	
Scheme:	weka.classifiers.functions.Logistic -R 1.0E-8 -M -1 -num-decimal-places 4	0.984	0.003	0.984	0.984	1	2			##	1	0	0	|	a	=	1
Scheme:	weka.classifiers.functions.Logistic -R 1.0E-8 -M -1 -num-decimal-places 4	1	0	1	1	1	3			1	60	0	0	|	b	=	2
Scheme:	weka.classifiers.functions.Logistic -R 1.0E-8 -M -1 -num-decimal-places 4	1	0	1	1	1	999			0	0	24	0	|	c	=	3
Scheme:	weka.classifiers.functions.Logistic -R 1.0E-8 -M -1 -num-decimal-places 4	0.995	0.008	0.995	0.995	1	Weighted ave.	99.54	0.464	0	0	0	#	|	d	=	##
Scheme:	weka.classifiers.functions.MultilayerPerceptron -L 0.3 -M 0.2 -N 500 -V 0 -S 0 -E 20 -H a	0.994	0.062	0.982	0.988	0.9	1			a	b	c	d	<--	classified	as	
Scheme:	weka.classifiers.functions.MultilayerPerceptron -L 0.3 -M 0.2 -N 500 -V 0 -S 0 -E 20 -H a	0.984	0.005	0.968	0.976	1	2			##	2	0	0	|	a	=	1
Scheme:	weka.classifiers.functions.MultilayerPerceptron -L 0.3 -M 0.2 -N 500 -V 0 -S 0 -E 20 -H a	0.792	0	1	0.884	0.9	3			1	60	0	0	|	b	=	2
Scheme:	weka.classifiers.functions.MultilayerPerceptron -L 0.3 -M 0.2 -N 500 -V 0 -S 0 -E 20 -H a	1	0	1	1	1	999			5	0	19	0	|	c	=	3
Scheme:	weka.classifiers.functions.MultilayerPerceptron -L 0.3 -M 0.2 -N 500 -V 0 -S 0 -E 20 -H a	0.981	0.049	0.982	0.981	0.9	Weighted ave.	98.14	1.8561	0	0	0	#	|	d	=	##
Scheme:	weka.classifiers.functions.MultilayerPerceptron -L 0.3 -M 0.2 -N 500 -V 0 -S 0 -E 20 -H t	0.994	0.072	0.979	0.987	0.9	1			a	b	c	d	<--	classified	as	
Scheme:	weka.classifiers.functions.MultilayerPerceptron -L 0.3 -M 0.2 -N 500 -V 0 -S 0 -E 20 -H t	0.967	0.005	0.967	0.967	1	2			##	2	0	0	|	a	=	1
Scheme:	weka.classifiers.functions.MultilayerPerceptron -L 0.3 -M 0.2 -N 500 -V 0 -S 0 -E 20 -H t	0.792	0	1	0.884	0.9	3			2	59	0	0	|	b	=	2
Scheme:	weka.classifiers.functions.MultilayerPerceptron -L 0.3 -M 0.2 -N 500 -V 0 -S 0 -E 20 -H t	1	0	1	1	1	999			5	0	19	0	|	c	=	3
Scheme:	weka.classifiers.functions.MultilayerPerceptron -L 0.3 -M 0.2 -N 500 -V 0 -S 0 -E 20 -H t	0.979	0.057	0.979	0.979	0.9	Weighted ave.	97.91	2.0882	0	0	0	#	|	d	=	##
Scheme:	weka.classifiers.functions.SimpleLogistic -I 0 -M 500 -H 50 -W 0.0	0.997	0.289	0.922	0.958	0.8	1			a	b	c	d	<--	classified	as	
Scheme:	weka.classifiers.functions.SimpleLogistic -I 0 -M 500 -H 50 -W 0.0	0.754	0.008	0.939	0.836	0.8	2			##	1	0	0	|	a	=	1
Scheme:	weka.classifiers.functions.SimpleLogistic -I 0 -M 500 -H 50 -W 0.0	0.625	0	1	0.769	0.8	3			15	46	0	0	|	b	=	2
Scheme:	weka.classifiers.functions.SimpleLogistic -I 0 -M 500 -H 50 -W 0.0	0.5	0	1	0.667	0.7	999			7	2	15	0	|	c	=	3
Scheme:	weka.classifiers.functions.SimpleLogistic -I 0 -M 500 -H 50 -W 0.0	0.928	0.225	0.931	0.922	0.8	Weighted ave.	92.81	7.1926	6	0	0	6	|	d	=	##
Scheme:	weka.classifiers.functions.SMO -C 1.0 -L 0.001 -P 1.0E-12 -N 0 -V -1 -W 1 -K "weka.classifiers.functions.supportVector.PolyKernel -E 1.0 -C 250007" -calibrator "weka.classifiers.functions.Logistic -R 1.0E-8 -M -1 -num-decimal-places 4"	0.997	0.567	0.858	0.922	0.6	1			a	b	c	d	<--	classified	as	
Scheme:	weka.classifiers.functions.SMO -C 1.0 -L 0.001 -P 1.0E-12 -N 0 -V -1 -W 1 -K "weka.classifiers.functions.supportVector.PolyKernel -E 1.0 -C 250007" -calibrator "weka.classifiers.functions.Logistic -R 1.0E-8 -M -1 -num-decimal-places 4"	0.41	0.016	0.806	0.543	0.5	2			##	1	0	0	|	a	=	1
Scheme:	weka.classifiers.functions.SMO -C 1.0 -L 0.001 -P 1.0E-12 -N 0 -V -1 -W 1 -K "weka.classifiers.functions.supportVector.PolyKernel -E 1.0 -C 250007" -calibrator "weka.classifiers.functions.Logistic -R 1.0E-8 -M -1 -num-decimal-places 4"	0	0	?	?	?	3			36	25	0	0	|	b	=	2
Scheme:	weka.classifiers.functions.SMO -C 1.0 -L 0.001 -P 1.0E-12 -N 0 -V -1 -W 1 -K "weka.classifiers.functions.supportVector.PolyKernel -E 1.0 -C 250007" -calibrator "weka.classifiers.functions.Logistic -R 1.0E-8 -M -1 -num-decimal-places 4"	1	0	1	1	1	999			19	5	0	0	|	c	=	3
Scheme:	weka.classifiers.functions.SMO -C 1.0 -L 0.001 -P 1.0E-12 -N 0 -V -1 -W 1 -K "weka.classifiers.functions.supportVector.PolyKernel -E 1.0 -C 250007" -calibrator "weka.classifiers.functions.Logistic -R 1.0E-8 -M -1 -num-decimal-places 4"	0.858	0.442	?	?	?	Weighted ave.	85.85	14.153	0	0	0	#	|	d	=	##
Scheme:	weka.classifiers.lazy.IBk -K 1 -W 0 -A "weka.core.neighboursearch.LinearNNSearch -A \"weka.core.EuclideanDistance -R first-last\""	0.997	0.01	0.997	0.997	1	1			a	b	c	d	<--	classified	as	
Scheme:	weka.classifiers.lazy.IBk -K 1 -W 0 -A "weka.core.neighboursearch.LinearNNSearch -A \"weka.core.EuclideanDistance -R first-last\""	0.984	0.003	0.984	0.984	1	2			##	1	0	0	|	a	=	1
Scheme:	weka.classifiers.lazy.IBk -K 1 -W 0 -A "weka.core.neighboursearch.LinearNNSearch -A \"weka.core.EuclideanDistance -R first-last\""	1	0	1	1	1	3			1	60	0	0	|	b	=	2
Scheme:	weka.classifiers.lazy.IBk -K 1 -W 0 -A "weka.core.neighboursearch.LinearNNSearch -A \"weka.core.EuclideanDistance -R first-last\""	1	0	1	1	1	999			0	0	24	0	|	c	=	3
Scheme:	weka.classifiers.lazy.IBk -K 1 -W 0 -A "weka.core.neighboursearch.LinearNNSearch -A \"weka.core.EuclideanDistance -R first-last\""	0.995	0.008	0.995	0.995	1	Weighted ave.	99.54	0.464	0	0	0	#	|	d	=	##
Scheme:	weka.classifiers.lazy.KStar -B 20 -M a	1	0.01	0.997	0.999	1	1			a	b	c	d	<--	classified	as	
Scheme:	weka.classifiers.lazy.KStar -B 20 -M a	0.984	0	1	0.992	1	2			##	0	0	0	|	a	=	1
Scheme:	weka.classifiers.lazy.KStar -B 20 -M a	1	0	1	1	1	3			1	60	0	0	|	b	=	2
Scheme:	weka.classifiers.lazy.KStar -B 20 -M a	1	0	1	1	1	999			0	0	24	0	|	c	=	3
Scheme:	weka.classifiers.lazy.KStar -B 20 -M a	0.998	0.008	0.998	0.998	1	Weighted ave.	99.77	0.232	0	0	0	#	|	d	=	##
Scheme:	weka.classifiers.lazy.LWL -U 0 -K -1 -A "weka.core.neighboursearch.LinearNNSearch -A \"weka.core.EuclideanDistance -R first-last\"" -W weka.classifiers.trees.DecisionStump	1	0.794	0.813	0.897	0.4	1			a	b	c	d	<--	classified	as	
Scheme:	weka.classifiers.lazy.LWL -U 0 -K -1 -A "weka.core.neighboursearch.LinearNNSearch -A \"weka.core.EuclideanDistance -R first-last\"" -W weka.classifiers.trees.DecisionStump	0.049	0.005	0.6	0.091	0.1	2			##	0	0	0	|	a	=	1
Scheme:	weka.classifiers.lazy.LWL -U 0 -K -1 -A "weka.core.neighboursearch.LinearNNSearch -A \"weka.core.EuclideanDistance -R first-last\"" -W weka.classifiers.trees.DecisionStump	0.125	0	1	0.222	0.3	3			58	3	0	0	|	b	=	2
Scheme:	weka.classifiers.lazy.LWL -U 0 -K -1 -A "weka.core.neighboursearch.LinearNNSearch -A \"weka.core.EuclideanDistance -R first-last\"" -W weka.classifiers.trees.DecisionStump	1	0	1	1	1	999			19	2	3	0	|	c	=	3
Scheme:	weka.classifiers.lazy.LWL -U 0 -K -1 -A "weka.core.neighboursearch.LinearNNSearch -A \"weka.core.EuclideanDistance -R first-last\"" -W weka.classifiers.trees.DecisionStump	0.817	0.616	0.798	0.748	0.4	Weighted ave.	81.67	18.33	0	0	0	#	|	d	=	##
Scheme:	weka.classifiers.meta.AdaBoostM1 -P 100 -S 1 -I 10 -W weka.classifiers.trees.DecisionStump	1	0.876	0.797	0.887	0.3	1			a	b	c	d	<--	classified	as	
Scheme:	weka.classifiers.meta.AdaBoostM1 -P 100 -S 1 -I 10 -W weka.classifiers.trees.DecisionStump	0	0	?	?	?	2			##	0	0	0	|	a	=	1
Scheme:	weka.classifiers.meta.AdaBoostM1 -P 100 -S 1 -I 10 -W weka.classifiers.trees.DecisionStump	0	0	?	?	?	3			61	0	0	0	|	b	=	2
Scheme:	weka.classifiers.meta.AdaBoostM1 -P 100 -S 1 -I 10 -W weka.classifiers.trees.DecisionStump	1	0	1	1	1	999			24	0	0	0	|	c	=	3
Scheme:	weka.classifiers.meta.AdaBoostM1 -P 100 -S 1 -I 10 -W weka.classifiers.trees.DecisionStump	0.803	0.679	?	?	?	Weighted ave.	80.28	19.722	0	0	0	#	|	d	=	##
Scheme:	weka.classifiers.meta.AdaBoostM1 -P 100 -S 1 -I 10 -W weka.classifiers.trees.RandomForest -- -P 100 -I 100 -num-slots 1 -K 0 -M 1.0 -V 0.001 -S 1	1	0.01	0.997	0.999	1	1			a	b	c	d	<--	classified	as	
Scheme:	weka.classifiers.meta.AdaBoostM1 -P 100 -S 1 -I 10 -W weka.classifiers.trees.RandomForest -- -P 100 -I 100 -num-slots 1 -K 0 -M 1.0 -V 0.001 -S 1	0.984	0	1	0.992	1	2			##	0	0	0	|	a	=	1
Scheme:	weka.classifiers.meta.AdaBoostM1 -P 100 -S 1 -I 10 -W weka.classifiers.trees.RandomForest -- -P 100 -I 100 -num-slots 1 -K 0 -M 1.0 -V 0.001 -S 1	1	0	1	1	1	3			1	60	0	0	|	b	=	2
Scheme:	weka.classifiers.meta.AdaBoostM1 -P 100 -S 1 -I 10 -W weka.classifiers.trees.RandomForest -- -P 100 -I 100 -num-slots 1 -K 0 -M 1.0 -V 0.001 -S 1	1	0	1	1	1	999			0	0	24	0	|	c	=	3
Scheme:	weka.classifiers.meta.AdaBoostM1 -P 100 -S 1 -I 10 -W weka.classifiers.trees.RandomForest -- -P 100 -I 100 -num-slots 1 -K 0 -M 1.0 -V 0.001 -S 1	0.998	0.008	0.998	0.998	1	Weighted ave.	99.77	0.232	0	0	0	#	|	d	=	##
Scheme:	weka.classifiers.meta.AttributeSelectedClassifier -E "weka.attributeSelection.CfsSubsetEval -P 1 -E 1" -S "weka.attributeSelection.BestFirst -D 1 -N 5" -W weka.classifiers.trees.J48 -- -C 0.25 -M 2	0.994	0.01	0.997	0.996	1	1			a	b	c	d	<--	classified	as	
Scheme:	weka.classifiers.meta.AttributeSelectedClassifier -E "weka.attributeSelection.CfsSubsetEval -P 1 -E 1" -S "weka.attributeSelection.BestFirst -D 1 -N 5" -W weka.classifiers.trees.J48 -- -C 0.25 -M 2	0.984	0.005	0.968	0.976	1	2			##	2	0	0	|	a	=	1
Scheme:	weka.classifiers.meta.AttributeSelectedClassifier -E "weka.attributeSelection.CfsSubsetEval -P 1 -E 1" -S "weka.attributeSelection.BestFirst -D 1 -N 5" -W weka.classifiers.trees.J48 -- -C 0.25 -M 2	1	0	1	1	1	3			1	60	0	0	|	b	=	2
Scheme:	weka.classifiers.meta.AttributeSelectedClassifier -E "weka.attributeSelection.CfsSubsetEval -P 1 -E 1" -S "weka.attributeSelection.BestFirst -D 1 -N 5" -W weka.classifiers.trees.J48 -- -C 0.25 -M 2	1	0	1	1	1	999			0	0	24	0	|	c	=	3
Scheme:	weka.classifiers.meta.AttributeSelectedClassifier -E "weka.attributeSelection.CfsSubsetEval -P 1 -E 1" -S "weka.attributeSelection.BestFirst -D 1 -N 5" -W weka.classifiers.trees.J48 -- -C 0.25 -M 2	0.993	0.009	0.993	0.993	1	Weighted ave.	99.3	0.6961	0	0	0	#	|	d	=	##
Scheme:	weka.classifiers.meta.Bagging -P 100 -S 1 -num-slots 1 -I 10 -W weka.classifiers.trees.REPTree -- -M 2 -V 0.001 -N 3 -S 1 -L -1 -I 0.0	1	0.062	0.982	0.991	1	1			a	b	c	d	<--	classified	as	
Scheme:	weka.classifiers.meta.Bagging -P 100 -S 1 -num-slots 1 -I 10 -W weka.classifiers.trees.REPTree -- -M 2 -V 0.001 -N 3 -S 1 -L -1 -I 0.0	0.967	0	1	0.983	1	2			##	0	0	0	|	a	=	1
Scheme:	weka.classifiers.meta.Bagging -P 100 -S 1 -num-slots 1 -I 10 -W weka.classifiers.trees.REPTree -- -M 2 -V 0.001 -N 3 -S 1 -L -1 -I 0.0	0.833	0	1	0.909	0.9	3			2	59	0	0	|	b	=	2
Scheme:	weka.classifiers.meta.Bagging -P 100 -S 1 -num-slots 1 -I 10 -W weka.classifiers.trees.REPTree -- -M 2 -V 0.001 -N 3 -S 1 -L -1 -I 0.0	1	0	1	1	1	999			4	0	20	0	|	c	=	3
Scheme:	weka.classifiers.meta.Bagging -P 100 -S 1 -num-slots 1 -I 10 -W weka.classifiers.trees.REPTree -- -M 2 -V 0.001 -N 3 -S 1 -L -1 -I 0.0	0.986	0.048	0.986	0.986	1	Weighted ave.	98.61	1.3921	0	0	0	#	|	d	=	##
Scheme:	weka.classifiers.meta.ClassificationViaRegression -W weka.classifiers.trees.M5P -- -M 4.0 -num-decimal-places 4	1	0.103	0.971	0.985	0.9	1			a	b	c	d	<--	classified	as	
Scheme:	weka.classifiers.meta.ClassificationViaRegression -W weka.classifiers.trees.M5P -- -M 4.0 -num-decimal-places 4	0.918	0	1	0.957	1	2			##	0	0	0	|	a	=	1
Scheme:	weka.classifiers.meta.ClassificationViaRegression -W weka.classifiers.trees.M5P -- -M 4.0 -num-decimal-places 4	0.792	0	1	0.884	0.9	3			5	56	0	0	|	b	=	2
Scheme:	weka.classifiers.meta.ClassificationViaRegression -W weka.classifiers.trees.M5P -- -M 4.0 -num-decimal-places 4	1	0	1	1	1	999			5	0	19	0	|	c	=	3
Scheme:	weka.classifiers.meta.ClassificationViaRegression -W weka.classifiers.trees.M5P -- -M 4.0 -num-decimal-places 4	0.977	0.08	0.977	0.976	0.9	Weighted ave.	97.68	2.3202	0	0	0	#	|	d	=	##
Scheme:	weka.classifiers.meta.CVParameterSelection -X 10 -S 1 -W weka.classifiers.rules.ZeroR	1	1	0.775	0.873	?	1			a	b	c	d	<--	classified	as	
Scheme:	weka.classifiers.meta.CVParameterSelection -X 10 -S 1 -W weka.classifiers.rules.ZeroR	0	0	?	?	?	2			##	0	0	0	|	a	=	1
Scheme:	weka.classifiers.meta.CVParameterSelection -X 10 -S 1 -W weka.classifiers.rules.ZeroR	0	0	?	?	?	3			61	0	0	0	|	b	=	2
Scheme:	weka.classifiers.meta.CVParameterSelection -X 10 -S 1 -W weka.classifiers.rules.ZeroR	0	0	?	?	?	999			24	0	0	0	|	c	=	3
Scheme:	weka.classifiers.meta.CVParameterSelection -X 10 -S 1 -W weka.classifiers.rules.ZeroR	0.775	0.775	?	?	?	Weighted ave.	77.49	22.506	12	0	0	0	|	d	=	##
Scheme:	weka.classifiers.meta.FilteredClassifier -F "weka.filters.supervised.attribute.Discretize -R first-last -precision 6" -S 1 -W weka.classifiers.trees.J48 -- -C 0.25 -M 2	1	0.062	0.982	0.991	1	1			a	b	c	d	<--	classified	as	
Scheme:	weka.classifiers.meta.FilteredClassifier -F "weka.filters.supervised.attribute.Discretize -R first-last -precision 6" -S 1 -W weka.classifiers.trees.J48 -- -C 0.25 -M 2	0.902	0	1	0.948	0.9	2			##	0	0	0	|	a	=	1
Scheme:	weka.classifiers.meta.FilteredClassifier -F "weka.filters.supervised.attribute.Discretize -R first-last -precision 6" -S 1 -W weka.classifiers.trees.J48 -- -C 0.25 -M 2	1	0	1	1	1	3			6	55	0	0	|	b	=	2
Scheme:	weka.classifiers.meta.FilteredClassifier -F "weka.filters.supervised.attribute.Discretize -R first-last -precision 6" -S 1 -W weka.classifiers.trees.J48 -- -C 0.25 -M 2	1	0	1	1	1	999			0	0	24	0	|	c	=	3
Scheme:	weka.classifiers.meta.FilteredClassifier -F "weka.filters.supervised.attribute.Discretize -R first-last -precision 6" -S 1 -W weka.classifiers.trees.J48 -- -C 0.25 -M 2	0.986	0.048	0.986	0.986	1	Weighted ave.	98.61	1.3921	0	0	0	#	|	d	=	##
Scheme:	weka.classifiers.meta.IterativeClassifierOptimizer -W weka.classifiers.meta.LogitBoost -L 50 -P 1 -E 1 -I 1 -F 10 -R 1 -percentage 0.0 -metric RMSE -S 1 -- -P 100 -L -1.7976931348623157E308 -H 1.0 -Z 3.0 -O 1 -E 1 -S 1 -I 10 -W weka.classifiers.trees.DecisionStump	1	0.093	0.974	0.987	0.9	1			a	b	c	d	<--	classified	as	
Scheme:	weka.classifiers.meta.IterativeClassifierOptimizer -W weka.classifiers.meta.LogitBoost -L 50 -P 1 -E 1 -I 1 -F 10 -R 1 -percentage 0.0 -metric RMSE -S 1 -- -P 100 -L -1.7976931348623157E308 -H 1.0 -Z 3.0 -O 1 -E 1 -S 1 -I 10 -W weka.classifiers.trees.DecisionStump	0.852	0	1	0.92	0.9	2			##	0	0	0	|	a	=	1
Scheme:	weka.classifiers.meta.IterativeClassifierOptimizer -W weka.classifiers.meta.LogitBoost -L 50 -P 1 -E 1 -I 1 -F 10 -R 1 -percentage 0.0 -metric RMSE -S 1 -- -P 100 -L -1.7976931348623157E308 -H 1.0 -Z 3.0 -O 1 -E 1 -S 1 -I 10 -W weka.classifiers.trees.DecisionStump	1	0	1	1	1	3			9	52	0	0	|	b	=	2
Scheme:	weka.classifiers.meta.IterativeClassifierOptimizer -W weka.classifiers.meta.LogitBoost -L 50 -P 1 -E 1 -I 1 -F 10 -R 1 -percentage 0.0 -metric RMSE -S 1 -- -P 100 -L -1.7976931348623157E308 -H 1.0 -Z 3.0 -O 1 -E 1 -S 1 -I 10 -W weka.classifiers.trees.DecisionStump	1	0	1	1	1	999			0	0	24	0	|	c	=	3
Scheme:	weka.classifiers.meta.IterativeClassifierOptimizer -W weka.classifiers.meta.LogitBoost -L 50 -P 1 -E 1 -I 1 -F 10 -R 1 -percentage 0.0 -metric RMSE -S 1 -- -P 100 -L -1.7976931348623157E308 -H 1.0 -Z 3.0 -O 1 -E 1 -S 1 -I 10 -W weka.classifiers.trees.DecisionStump	0.979	0.072	0.98	0.978	0.9	Weighted ave.	97.91	2.0882	0	0	0	#	|	d	=	##
Scheme:	weka.classifiers.meta.IterativeClassifierOptimizer -W weka.classifiers.meta.LogitBoost -L 50 -P 1 -E 1 -I 1 -F 10 -R 1 -percentage 0.0 -metric RMSE -S 1 -- -P 100 -L -1.7976931348623157E308 -H 1.0 -Z 3.0 -O 1 -E 1 -S 1 -I 10 -W weka.classifiers.trees.RandomForest -- -P 100 -I 100 -num-slots 1	1	0.01	0.997	0.999	1	1			a	b	c	d	<--	classified	as	
Scheme:	weka.classifiers.meta.IterativeClassifierOptimizer -W weka.classifiers.meta.LogitBoost -L 50 -P 1 -E 1 -I 1 -F 10 -R 1 -percentage 0.0 -metric RMSE -S 1 -- -P 100 -L -1.7976931348623157E308 -H 1.0 -Z 3.0 -O 1 -E 1 -S 1 -I 10 -W weka.classifiers.trees.RandomForest -- -P 100 -I 100 -num-slots 1	0.984	0	1	0.992	1	2			##	0	0	0	|	a	=	1
Scheme:	weka.classifiers.meta.IterativeClassifierOptimizer -W weka.classifiers.meta.LogitBoost -L 50 -P 1 -E 1 -I 1 -F 10 -R 1 -percentage 0.0 -metric RMSE -S 1 -- -P 100 -L -1.7976931348623157E308 -H 1.0 -Z 3.0 -O 1 -E 1 -S 1 -I 10 -W weka.classifiers.trees.RandomForest -- -P 100 -I 100 -num-slots 1	1	0	1	1	1	3			1	60	0	0	|	b	=	2
Scheme:	weka.classifiers.meta.IterativeClassifierOptimizer -W weka.classifiers.meta.LogitBoost -L 50 -P 1 -E 1 -I 1 -F 10 -R 1 -percentage 0.0 -metric RMSE -S 1 -- -P 100 -L -1.7976931348623157E308 -H 1.0 -Z 3.0 -O 1 -E 1 -S 1 -I 10 -W weka.classifiers.trees.RandomForest -- -P 100 -I 100 -num-slots 1	1	0	1	1	1	999			0	0	24	0	|	c	=	3
Scheme:	weka.classifiers.meta.IterativeClassifierOptimizer -W weka.classifiers.meta.LogitBoost -L 50 -P 1 -E 1 -I 1 -F 10 -R 1 -percentage 0.0 -metric RMSE -S 1 -- -P 100 -L -1.7976931348623157E308 -H 1.0 -Z 3.0 -O 1 -E 1 -S 1 -I 10 -W weka.classifiers.trees.RandomForest -- -P 100 -I 100 -num-slots 1	0.998	0.008	0.998	0.998	1	Weighted ave.	99.77	0.232	0	0	0	#	|	d	=	##
Scheme:	weka.classifiers.meta.LogitBoost -P 100 -L -1.7976931348623157E308 -H 1.0 -Z 3.0 -O 1 -E 1 -S 1 -I 10 -W weka.classifiers.trees.DecisionStump	1	0.093	0.974	0.987	0.9	1			a	b	c	d	<--	classified	as	
Scheme:	weka.classifiers.meta.LogitBoost -P 100 -L -1.7976931348623157E308 -H 1.0 -Z 3.0 -O 1 -E 1 -S 1 -I 10 -W weka.classifiers.trees.DecisionStump	0.852	0	1	0.92	0.9	2			##	0	0	0	|	a	=	1

Scheme:	weka.classifiers.meta.LogitBoost -P 100 -L -1.7976931348623157E308 -H 1.0 -Z 3.0 -O 1 -E 1 -S 1 -I 10 -W weka.classifiers.trees.DecisionStump	1	0	1	1	1	3			9	52	0	0	|	b	=	2
Scheme:	weka.classifiers.meta.LogitBoost -P 100 -L -1.7976931348623157E308 -H 1.0 -Z 3.0 -O 1 -E 1 -S 1 -I 10 -W weka.classifiers.trees.DecisionStump	1	0	1	1	1	999			0	0	24	0	|	c	=	3
Scheme:	weka.classifiers.meta.LogitBoost -P 100 -L -1.7976931348623157E308 -H 1.0 -Z 3.0 -O 1 -E 1 -S 1 -I 10 -W weka.classifiers.trees.DecisionStump	0.979	0.072	0.98	0.978	0.9	Weighted ave.	97.91	2.0882	0	0	0	#	|	d	=	##
Scheme:	weka.classifiers.meta.LogitBoost -P 100 -L -1.7976931348623157E308 -H 1.0 -Z 3.0 -O 1 -E 1 -S 1 -I 10 -W weka.classifiers.trees.RandomForest -- -P 100 -I 100 -num-slots 1 -K 0 -M 1.0 -V 0.001 -S 1	1	0.01	0.997	0.999	1	1			a	b	c	d	<--	classified	as	
Scheme:	weka.classifiers.meta.LogitBoost -P 100 -L -1.7976931348623157E308 -H 1.0 -Z 3.0 -O 1 -E 1 -S 1 -I 10 -W weka.classifiers.trees.RandomForest -- -P 100 -I 100 -num-slots 1 -K 0 -M 1.0 -V 0.001 -S 1	0.984	0	1	0.992	1	2			##	0	0	0	|	a	=	1
Scheme:	weka.classifiers.meta.LogitBoost -P 100 -L -1.7976931348623157E308 -H 1.0 -Z 3.0 -O 1 -E 1 -S 1 -I 10 -W weka.classifiers.trees.RandomForest -- -P 100 -I 100 -num-slots 1 -K 0 -M 1.0 -V 0.001 -S 1	1	0	1	1	1	3			1	60	0	0	|	b	=	2
Scheme:	weka.classifiers.meta.LogitBoost -P 100 -L -1.7976931348623157E308 -H 1.0 -Z 3.0 -O 1 -E 1 -S 1 -I 10 -W weka.classifiers.trees.RandomForest -- -P 100 -I 100 -num-slots 1 -K 0 -M 1.0 -V 0.001 -S 1	1	0	1	1	1	999			0	0	24	0	|	c	=	3
Scheme:	weka.classifiers.meta.LogitBoost -P 100 -L -1.7976931348623157E308 -H 1.0 -Z 3.0 -O 1 -E 1 -S 1 -I 10 -W weka.classifiers.trees.RandomForest -- -P 100 -I 100 -num-slots 1 -K 0 -M 1.0 -V 0.001 -S 1	0.998	0.008	0.998	0.998	1	Weighted ave.	99.77	0.232	0	0	0	#	|	d	=	##
Scheme:	weka.classifiers.meta.LogitBoost -P 100 -L -1.7976931348623157E308 -H 1.0 -Z 3.0 -O 1 -E 1 -S 1 -I 10 -W weka.classifiers.trees.RandomForest -- -P 100 -I 1000 -num-slots 1 -K 0 -M 1.0 -V 0.001 -S 1	1	0.01	0.997	0.999	1	1			a	b	c	d	<--	classified	as	
Scheme:	weka.classifiers.meta.LogitBoost -P 100 -L -1.7976931348623157E308 -H 1.0 -Z 3.0 -O 1 -E 1 -S 1 -I 10 -W weka.classifiers.trees.RandomForest -- -P 100 -I 1000 -num-slots 1 -K 0 -M 1.0 -V 0.001 -S 1	0.984	0	1	0.992	1	2			##	0	0	0	|	a	=	1
Scheme:	weka.classifiers.meta.LogitBoost -P 100 -L -1.7976931348623157E308 -H 1.0 -Z 3.0 -O 1 -E 1 -S 1 -I 10 -W weka.classifiers.trees.RandomForest -- -P 100 -I 1000 -num-slots 1 -K 0 -M 1.0 -V 0.001 -S 1	1	0	1	1	1	3			1	60	0	0	|	b	=	2
Scheme:	weka.classifiers.meta.LogitBoost -P 100 -L -1.7976931348623157E308 -H 1.0 -Z 3.0 -O 1 -E 1 -S 1 -I 10 -W weka.classifiers.trees.RandomForest -- -P 100 -I 1000 -num-slots 1 -K 0 -M 1.0 -V 0.001 -S 1	1	0	1	1	1	999			0	0	24	0	|	c	=	3
Scheme:	weka.classifiers.meta.LogitBoost -P 100 -L -1.7976931348623157E308 -H 1.0 -Z 3.0 -O 1 -E 1 -S 1 -I 10 -W weka.classifiers.trees.RandomForest -- -P 100 -I 1000 -num-slots 1 -K 0 -M 1.0 -V 0.001 -S 1	0.998	0.008	0.998	0.998	1	Weighted ave.	99.77	0.232	0	0	0	#	|	d	=	##
Scheme:	weka.classifiers.meta.MultiClassClassifier -M 0 -R 2.0 -S 1 -W weka.classifiers.functions.Logistic -- -R 1.0E-8 -M -1 -num-decimal-places 4	0.997	0.01	0.997	0.997	1	1			a	b	c	d	<--	classified	as	
Scheme:	weka.classifiers.meta.MultiClassClassifier -M 0 -R 2.0 -S 1 -W weka.classifiers.functions.Logistic -- -R 1.0E-8 -M -1 -num-decimal-places 4	0.984	0.003	0.984	0.984	1	2			##	1	0	0	|	a	=	1
Scheme:	weka.classifiers.meta.MultiClassClassifier -M 0 -R 2.0 -S 1 -W weka.classifiers.functions.Logistic -- -R 1.0E-8 -M -1 -num-decimal-places 4	1	0	1	1	1	3			1	60	0	0	|	b	=	2
Scheme:	weka.classifiers.meta.MultiClassClassifier -M 0 -R 2.0 -S 1 -W weka.classifiers.functions.Logistic -- -R 1.0E-8 -M -1 -num-decimal-places 4	1	0	1	1	1	999			0	0	24	0	|	c	=	3
Scheme:	weka.classifiers.meta.MultiClassClassifier -M 0 -R 2.0 -S 1 -W weka.classifiers.functions.Logistic -- -R 1.0E-8 -M -1 -num-decimal-places 4	0.995	0.008	0.995	0.995	1	Weighted ave.	99.54	0.464	0	0	0	#	|	d	=	##
Scheme:	weka.classifiers.meta.MultiClassClassifierUpdateable -M 0 -R 2.0 -S 1 -W weka.classifiers.functions.SGD -- -F 0 -L 0.01 -R 1.0E-4 -E 500 -C 0.001 -S 1	0.997	0.412	0.893	0.942	0.7	1			a	b	c	d	<--	classified	as	
Scheme:	weka.classifiers.meta.MultiClassClassifierUpdateable -M 0 -R 2.0 -S 1 -W weka.classifiers.functions.SGD -- -F 0 -L 0.01 -R 1.0E-4 -E 500 -C 0.001 -S 1	0.623	0.016	0.864	0.724	0.7	2			##	1	0	0	|	a	=	1
Scheme:	weka.classifiers.meta.MultiClassClassifierUpdateable -M 0 -R 2.0 -S 1 -W weka.classifiers.functions.SGD -- -F 0 -L 0.01 -R 1.0E-4 -E 500 -C 0.001 -S 1	0.083	0	1	0.154	0.3	3			23	38	0	0	|	b	=	2
Scheme:	weka.classifiers.meta.MultiClassClassifierUpdateable -M 0 -R 2.0 -S 1 -W weka.classifiers.functions.SGD -- -F 0 -L 0.01 -R 1.0E-4 -E 500 -C 0.001 -S 1	1	0	1	1	1	999			17	5	2	0	|	c	=	3
Scheme:	weka.classifiers.meta.MultiClassClassifierUpdateable -M 0 -R 2.0 -S 1 -W weka.classifiers.functions.SGD -- -F 0 -L 0.01 -R 1.0E-4 -E 500 -C 0.001 -S 1	0.893	0.322	0.898	0.869	0.7	Weighted ave.	89.33	10.673	0	0	0	#	|	d	=	##
Scheme:	weka.classifiers.meta.MultiScheme -X 0 -S 1 -B "weka.classifiers.trees.RandomForest -P 100 -I 100 -num-slots 1 -K 0 -M 1.0 -V 0.001 -S 1" -B "weka.classifiers.functions.MultilayerPerceptron -L 0.3 -M 0.2 -N 500 -V 0 -S 0 -E 20 -H a"	1	0.01	0.997	0.999	1	1			a	b	c	d	<--	classified	as	
Scheme:	weka.classifiers.meta.MultiScheme -X 0 -S 1 -B "weka.classifiers.trees.RandomForest -P 100 -I 100 -num-slots 1 -K 0 -M 1.0 -V 0.001 -S 1" -B "weka.classifiers.functions.MultilayerPerceptron -L 0.3 -M 0.2 -N 500 -V 0 -S 0 -E 20 -H a"	0.984	0	1	0.992	1	2			##	0	0	0	|	a	=	1
Scheme:	weka.classifiers.meta.MultiScheme -X 0 -S 1 -B "weka.classifiers.trees.RandomForest -P 100 -I 100 -num-slots 1 -K 0 -M 1.0 -V 0.001 -S 1" -B "weka.classifiers.functions.MultilayerPerceptron -L 0.3 -M 0.2 -N 500 -V 0 -S 0 -E 20 -H a"	1	0	1	1	1	3			1	60	0	0	|	b	=	2
Scheme:	weka.classifiers.meta.MultiScheme -X 0 -S 1 -B "weka.classifiers.trees.RandomForest -P 100 -I 100 -num-slots 1 -K 0 -M 1.0 -V 0.001 -S 1" -B "weka.classifiers.functions.MultilayerPerceptron -L 0.3 -M 0.2 -N 500 -V 0 -S 0 -E 20 -H a"	1	0	1	1	1	999			0	0	24	0	|	c	=	3
Scheme:	weka.classifiers.meta.MultiScheme -X 0 -S 1 -B "weka.classifiers.trees.RandomForest -P 100 -I 100 -num-slots 1 -K 0 -M 1.0 -V 0.001 -S 1" -B "weka.classifiers.functions.MultilayerPerceptron -L 0.3 -M 0.2 -N 500 -V 0 -S 0 -E 20 -H a"	0.998	0.008	0.998	0.998	1	Weighted ave.	99.77	0.232	0	0	0	#	|	d	=	##
Scheme:	weka.classifiers.meta.RandomCommittee -S 1 -num-slots 1 -I 10 -W weka.classifiers.trees.RandomTree -- -K 0 -M 1.0 -V 0.001 -S 1	1	0.01	0.997	0.999	1	1			a	b	c	d	<--	classified	as	
Scheme:	weka.classifiers.meta.RandomCommittee -S 1 -num-slots 1 -I 10 -W weka.classifiers.trees.RandomTree -- -K 0 -M 1.0 -V 0.001 -S 1	0.984	0	1	0.992	1	2			##	0	0	0	|	a	=	1
Scheme:	weka.classifiers.meta.RandomCommittee -S 1 -num-slots 1 -I 10 -W weka.classifiers.trees.RandomTree -- -K 0 -M 1.0 -V 0.001 -S 1	1	0	1	1	1	3			1	60	0	0	|	b	=	2
Scheme:	weka.classifiers.meta.RandomCommittee -S 1 -num-slots 1 -I 10 -W weka.classifiers.trees.RandomTree -- -K 0 -M 1.0 -V 0.001 -S 1	1	0	1	1	1	999			0	0	24	0	|	c	=	3
Scheme:	weka.classifiers.meta.RandomCommittee -S 1 -num-slots 1 -I 10 -W weka.classifiers.trees.RandomTree -- -K 0 -M 1.0 -V 0.001 -S 1	0.998	0.008	0.998	0.998	1	Weighted ave.	99.77	0.232	0	0	0	#	|	d	=	##
Scheme:	weka.classifiers.meta.RandomizableFilteredClassifier -F "weka.filters.unsupervised.attribute.RandomProjection -N 10 -R 42 -D Sparse1" -S 1 -W weka.classifiers.lazy.IBk -- -K 1 -W 0 -A "weka.core.neighboursearch.LinearNNSearch -A \"weka.core.EuclideanDistance -R first-last\""	1	0	1	1	1	1			a	b	c	d	<--	classified	as	
Scheme:	weka.classifiers.meta.RandomizableFilteredClassifier -F "weka.filters.unsupervised.attribute.RandomProjection -N 10 -R 42 -D Sparse1" -S 1 -W weka.classifiers.lazy.IBk -- -K 1 -W 0 -A "weka.core.neighboursearch.LinearNNSearch -A \"weka.core.EuclideanDistance -R first-last\""	1	0	1	1	1	2			##	0	0	0	|	a	=	1
Scheme:	weka.classifiers.meta.RandomizableFilteredClassifier -F "weka.filters.unsupervised.attribute.RandomProjection -N 10 -R 42 -D Sparse1" -S 1 -W weka.classifiers.lazy.IBk -- -K 1 -W 0 -A "weka.core.neighboursearch.LinearNNSearch -A \"weka.core.EuclideanDistance -R first-last\""	1	0	1	1	1	3			0	61	0	0	|	b	=	2
Scheme:	weka.classifiers.meta.RandomizableFilteredClassifier -F "weka.filters.unsupervised.attribute.RandomProjection -N 10 -R 42 -D Sparse1" -S 1 -W weka.classifiers.lazy.IBk -- -K 1 -W 0 -A "weka.core.neighboursearch.LinearNNSearch -A \"weka.core.EuclideanDistance -R first-last\""	1	0	1	1	1	999			0	0	24	0	|	c	=	3
Scheme:	weka.classifiers.meta.RandomizableFilteredClassifier -F "weka.filters.unsupervised.attribute.RandomProjection -N 10 -R 42 -D Sparse1" -S 1 -W weka.classifiers.lazy.IBk -- -K 1 -W 0 -A "weka.core.neighboursearch.LinearNNSearch -A \"weka.core.EuclideanDistance -R first-last\""	1	0	1	1	1	Weighted ave.	100	0	0	0	0	#	|	d	=	##
Scheme:	weka.classifiers.meta.RandomSubSpace -P 0.5 -S 1 -num-slots 1 -I 10 -W weka.classifiers.trees.REPTree -- -M 2 -V 0.001 -N 3 -S 1 -L -1 -I 0.0	1	0.01	0.997	0.999	1	1			a	b	c	d	<--	classified	as	
Scheme:	weka.classifiers.meta.RandomSubSpace -P 0.5 -S 1 -num-slots 1 -I 10 -W weka.classifiers.trees.REPTree -- -M 2 -V 0.001 -N 3 -S 1 -L -1 -I 0.0	0.984	0	1	0.992	1	2			##	0	0	0	|	a	=	1
Scheme:	weka.classifiers.meta.RandomSubSpace -P 0.5 -S 1 -num-slots 1 -I 10 -W weka.classifiers.trees.REPTree -- -M 2 -V 0.001 -N 3 -S 1 -L -1 -I 0.0	1	0	1	1	1	3			1	60	0	0	|	b	=	2
Scheme:	weka.classifiers.meta.RandomSubSpace -P 0.5 -S 1 -num-slots 1 -I 10 -W weka.classifiers.trees.REPTree -- -M 2 -V 0.001 -N 3 -S 1 -L -1 -I 0.0	1	0	1	1	1	999			0	0	24	0	|	c	=	3
Scheme:	weka.classifiers.meta.RandomSubSpace -P 0.5 -S 1 -num-slots 1 -I 10 -W weka.classifiers.trees.REPTree -- -M 2 -V 0.001 -N 3 -S 1 -L -1 -I 0.0	0.998	0.008	0.998	0.998	1	Weighted ave.	99.77	0.232	0	0	0	#	|	d	=	##
Scheme:	weka.classifiers.meta.Stacking -X 10 -M "weka.classifiers.trees.RandomForest -P 100 -I 100 -num-slots 1 -K 0 -M 1.0 -V 0.001 -S 1" -S 1 -num-slots 1 -B "weka.classifiers.rules.DecisionTable -X 1 -S \"weka.attributeSelection.BestFirst -D 1 -N 5\"" -B "weka.classifiers.functions.MultilayerPercept	0.994	0.041	0.988	0.991	1	1			a	b	c	d	<--	classified	as	
Scheme:	weka.classifiers.meta.Stacking -X 10 -M "weka.classifiers.trees.RandomForest -P 100 -I 100 -num-slots 1 -K 0 -M 1.0 -V 0.001 -S 1" -S 1 -num-slots 1 -B "weka.classifiers.rules.DecisionTable -X 1 -S \"weka.attributeSelection.BestFirst -D 1 -N 5\"" -B "weka.classifiers.functions.MultilayerPercept	0.934	0.005	0.966	0.95	0.9	2			##	2	0	0	|	a	=	1
Scheme:	weka.classifiers.meta.Stacking -X 10 -M "weka.classifiers.trees.RandomForest -P 100 -I 100 -num-slots 1 -K 0 -M 1.0 -V 0.001 -S 1" -S 1 -num-slots 1 -B "weka.classifiers.rules.DecisionTable -X 1 -S \"weka.attributeSelection.BestFirst -D 1 -N 5\"" -B "weka.classifiers.functions.MultilayerPercept	1	0	1	1	1	3			4	57	0	0	|	b	=	2
Scheme:	weka.classifiers.meta.Stacking -X 10 -M "weka.classifiers.trees.RandomForest -P 100 -I 100 -num-slots 1 -K 0 -M 1.0 -V 0.001 -S 1" -S 1 -num-slots 1 -B "weka.classifiers.rules.DecisionTable -X 1 -S \"weka.attributeSelection.BestFirst -D 1 -N 5\"" -B "weka.classifiers.functions.MultilayerPercept	1	0	1	1	1	999			0	0	24	0	|	c	=	3
Scheme:	weka.classifiers.meta.Stacking -X 10 -M "weka.classifiers.trees.RandomForest -P 100 -I 100 -num-slots 1 -K 0 -M 1.0 -V 0.001 -S 1" -S 1 -num-slots 1 -B "weka.classifiers.rules.DecisionTable -X 1 -S \"weka.attributeSelection.BestFirst -D 1 -N 5\"" -B "weka.classifiers.functions.MultilayerPercept	0.986	0.033	0.986	0.986	1	Weighted ave.	98.61	1.3921	0	0	0	#	|	d	=	##

Analysis OS:

Scheme		Recall	FP Rate	Precision	F-Measure	MCC	Class	Correct	Incorrect								
Scheme:	weka.classifiers.rules.ZeroR	1	1	0.749	0.857	?	1			a	b	c	d	<--	classified	as	
Scheme:	weka.classifiers.rules.ZeroR	0	0	?	?	?	2			##	0	0	0	|	a	=	1
Scheme:	weka.classifiers.rules.ZeroR	0	0	?	?	?	3			60	0	0	0	|	b	=	2
Scheme:	weka.classifiers.rules.ZeroR	0	0	?	?	?	999			36	0	0	0	|	c	=	3
Scheme:	weka.classifiers.rules.ZeroR	0.749	0.749	?	?	?	Weighted ave.	74.94	25.058	12	0	0	0	|	d	=	##
Scheme:	weka.classifiers.rules.DecisionTable -X 1 -S "weka.attributeSelection.BestFirst -D 1 -N 5"	1	0.037	0.988	0.994	1	1			a	b	c	d	<--	classified	as	
Scheme:	weka.classifiers.rules.DecisionTable -X 1 -S "weka.attributeSelection.BestFirst -D 1 -N 5"	0.967	0	1	0.983	1	2			##	0	0	0	|	a	=	1
Scheme:	weka.classifiers.rules.DecisionTable -X 1 -S "weka.attributeSelection.BestFirst -D 1 -N 5"	0.944	0	1	0.971	1	3			2	58	0	0	|	b	=	2
Scheme:	weka.classifiers.rules.DecisionTable -X 1 -S "weka.attributeSelection.BestFirst -D 1 -N 5"	1	0	1	1	1	999			2	0	34	0	|	c	=	3
Scheme:	weka.classifiers.rules.DecisionTable -X 1 -S "weka.attributeSelection.BestFirst -D 1 -N 5"	0.991	0.028	0.991	0.991	1	Weighted ave.	99.07	0.9281	0	0	0	#	|	d	=	##
Scheme:	weka.classifiers.rules.JRip -F 3 -N 2.0 -O 2 -S 1	0.994	0.009	0.997	0.995	1	1			a	b	c	d	<--	classified	as	
Scheme:	weka.classifiers.rules.JRip -F 3 -N 2.0 -O 2 -S 1	1	0	1	1	1	2			##	0	2	0	|	a	=	1
Scheme:	weka.classifiers.rules.JRip -F 3 -N 2.0 -O 2 -S 1	0.972	0.005	0.946	0.959	1	3			0	60	0	0	|	b	=	2
Scheme:	weka.classifiers.rules.JRip -F 3 -N 2.0 -O 2 -S 1	1	0	1	1	1	999			1	0	35	0	|	c	=	3
Scheme:	weka.classifiers.rules.JRip -F 3 -N 2.0 -O 2 -S 1	0.993	0.007	0.993	0.993	1	Weighted ave.	99.3	0.6961	0	0	0	#	|	d	=	##
Scheme:	weka.classifiers.rules.OneR -B 6	0.978	0.12	0.96	0.969	0.9	1			a	b	c	d	<--	classified	as	
Scheme:	weka.classifiers.rules.OneR -B 6	0.85	0.016	0.895	0.872	0.9	2			##	6	1	0	|	a	=	1
Scheme:	weka.classifiers.rules.OneR -B 6	0.778	0.025	0.737	0.757	0.7	3			0	51	9	0	|	b	=	2
Scheme:	weka.classifiers.rules.OneR -B 6	0.583	0	1	0.737	0.8	999			8	0	28	0	|	c	=	3
Scheme:	weka.classifiers.rules.OneR -B 6	0.933	0.095	0.934	0.932	0.9	Weighted ave.	93.27	6.7285	5	0	0	7	|	d	=	##
Scheme:	weka.classifiers.rules.PART -C 0.25 -M 2	1	0.009	0.997	0.998	1	1			a	b	c	d	<--	classified	as	
Scheme:	weka.classifiers.rules.PART -C 0.25 -M 2	1	0	1	1	1	2			##	0	0	0	|	a	=	1
Scheme:	weka.classifiers.rules.PART -C 0.25 -M 2	0.972	0	1	0.986	1	3			0	60	0	0	|	b	=	2
Scheme:	weka.classifiers.rules.PART -C 0.25 -M 2	1	0	1	1	1	999			1	0	35	0	|	c	=	3
Scheme:	weka.classifiers.rules.PART -C 0.25 -M 2	0.998	0.007	0.998	0.998	1	Weighted ave.	99.77	0.232	0	0	0	#	|	d	=	##
Scheme:	weka.classifiers.trees.RandomForest -P 100 -I 100 -num-slots 1 -K 0 -M 1.0 -V 0.001 -S 1	1	0	1	1	1	1			a	b	c	d	<--	classified	as	
Scheme:	weka.classifiers.trees.RandomForest -P 100 -I 100 -num-slots 1 -K 0 -M 1.0 -V 0.001 -S 1	1	0	1	1	1	2			##	0	0	0	|	a	=	1
Scheme:	weka.classifiers.trees.RandomForest -P 100 -I 100 -num-slots 1 -K 0 -M 1.0 -V 0.001 -S 1	1	0	1	1	1	3			0	60	0	0	|	b	=	2
Scheme:	weka.classifiers.trees.RandomForest -P 100 -I 100 -num-slots 1 -K 0 -M 1.0 -V 0.001 -S 1	1	0	1	1	1	999			0	0	36	0	|	c	=	3
Scheme:	weka.classifiers.trees.RandomForest -P 100 -I 100 -num-slots 1 -K 0 -M 1.0 -V 0.001 -S 1	1	0	1	1	1	Weighted ave.	100	0	0	0	0	#	|	d	=	##
Scheme:	weka.classifiers.trees.DecisionStump	1	0.889	0.771	0.871	0.3	1			a	b	c	d	<--	classified	as	
Scheme:	weka.classifiers.trees.DecisionStump	0	0	?	?	?	2			##	0	0	0	|	a	=	1
Scheme:	weka.classifiers.trees.DecisionStump	0	0	?	?	?	3			60	0	0	0	|	b	=	2
Scheme:	weka.classifiers.trees.DecisionStump	1	0	1	1	1	999			36	0	0	0	|	c	=	3
Scheme:	weka.classifiers.trees.DecisionStump	0.777	0.666	?	?	?	Weighted ave.	77.73	22.274	0	0	0	#	|	d	=	##
Scheme:	weka.classifiers.bayes.BayesNet -D -Q weka.classifiers.bayes.net.search.local.K2 -- -P 1 -S BAYES -E weka.classifiers.bayes.net.estimate.SimpleEstimator -- -A 0.5	0.994	0.019	0.994	0.994	1	1			a	b	c	d	<--	classified	as	
Scheme:	weka.classifiers.bayes.BayesNet -D -Q weka.classifiers.bayes.net.search.local.K2 -- -P 1 -S BAYES -E weka.classifiers.bayes.net.estimate.SimpleEstimator -- -A 0.5	0.933	0	1	0.966	1	2			##	0	2	0	|	a	=	1
Scheme:	weka.classifiers.bayes.BayesNet -D -Q weka.classifiers.bayes.net.search.local.K2 -- -P 1 -S BAYES -E weka.classifiers.bayes.net.estimate.SimpleEstimator -- -A 0.5	1	0.01	0.9	0.947	0.9	3			2	56	2	0	|	b	=	2
Scheme:	weka.classifiers.bayes.BayesNet -D -Q weka.classifiers.bayes.net.search.local.K2 -- -P 1 -S BAYES -E weka.classifiers.bayes.net.estimate.SimpleEstimator -- -A 0.5	1	0	1	1	1	999			0	0	36	0	|	c	=	3
Scheme:	weka.classifiers.bayes.BayesNet -D -Q weka.classifiers.bayes.net.search.local.K2 -- -P 1 -S BAYES -E weka.classifiers.bayes.net.estimate.SimpleEstimator -- -A 0.5	0.986	0.015	0.987	0.986	1	Weighted ave.	98.61	1.3921	0	0	0	#	|	d	=	##
Scheme:	weka.classifiers.bayes.NaiveBayes	0.192	0.028	0.954	0.32	0.2	1			a	b	c	d	<--	classified	as	
Scheme:	weka.classifiers.bayes.NaiveBayes	0.8	0.461	0.219	0.344	0.2	2			62	##	##	0	|	a	=	1
Scheme:	weka.classifiers.bayes.NaiveBayes	0.556	0.291	0.148	0.234	0.2	3			0	48	12	0	|	b	=	2
Scheme:	weka.classifiers.bayes.NaiveBayes	1	0	1	1	1	999			3	13	20	0	|	c	=	3
Scheme:	weka.classifiers.bayes.NaiveBayes	0.329	0.109	0.786	0.335	0.2	Weighted ave.	32.95	67.053	0	0	0	#	|	d	=	##
Scheme:	weka.classifiers.bayes.NaiveBayesMultinomial	0.176	0.046	0.919	0.296	0.2	1			a	b	c	d	<--	classified	as	
Scheme:	weka.classifiers.bayes.NaiveBayesMultinomial	0.8	0.59	0.18	0.294	0.2	2			57	##	48	#	|	a	=	1
Scheme:	weka.classifiers.bayes.NaiveBayesMultinomial	0.444	0.139	0.225	0.299	0.2	3			5	48	7	0	|	b	=	2
Scheme:	weka.classifiers.bayes.NaiveBayesMultinomial	1	0.045	0.387	0.558	0.6	999			0	20	16	0	|	c	=	3
Scheme:	weka.classifiers.bayes.NaiveBayesMultinomial	0.309	0.13	0.744	0.303	0.2	Weighted ave.	30.86	69.142	0	0	0	#	|	d	=	##
Scheme:	weka.classifiers.functions.Logistic -R 1.0E-8 -M -1 -num-decimal-places 4	0.994	0.065	0.979	0.986	0.9	1			a	b	c	d	<--	classified	as	
Scheme:	weka.classifiers.functions.Logistic -R 1.0E-8 -M -1 -num-decimal-places 4	0.883	0.005	0.964	0.922	0.9	2			##	2	0	0	|	a	=	1
Scheme:	weka.classifiers.functions.Logistic -R 1.0E-8 -M -1 -num-decimal-places 4	1	0	1	1	1	3			7	53	0	0	|	b	=	2
Scheme:	weka.classifiers.functions.Logistic -R 1.0E-8 -M -1 -num-decimal-places 4	1	0	1	1	1	999			0	0	36	0	|	c	=	3
Scheme:	weka.classifiers.functions.Logistic -R 1.0E-8 -M -1 -num-decimal-places 4	0.979	0.049	0.979	0.979	0.9	Weighted ave.	97.91	2.0882	0	0	0	#	|	d	=	##
Scheme:	weka.classifiers.functions.MultilayerPerceptron -L 0.3 -M 0.2 -N 500 -V 0 -S 0 -E 20 -H a	1	0.037	0.988	0.994	1	1			a	b	c	d	<--	classified	as	
Scheme:	weka.classifiers.functions.MultilayerPerceptron -L 0.3 -M 0.2 -N 500 -V 0 -S 0 -E 20 -H a	0.933	0.003	0.982	0.957	1	2			##	0	0	0	|	a	=	1
Scheme:	weka.classifiers.functions.MultilayerPerceptron -L 0.3 -M 0.2 -N 500 -V 0 -S 0 -E 20 -H a	0.917	0.005	0.943	0.93	0.9	3			2	56	2	0	|	b	=	2
Scheme:	weka.classifiers.functions.MultilayerPerceptron -L 0.3 -M 0.2 -N 500 -V 0 -S 0 -E 20 -H a	1	0	1	1	1	999			2	1	33	0	|	c	=	3
Scheme:	weka.classifiers.functions.MultilayerPerceptron -L 0.3 -M 0.2 -N 500 -V 0 -S 0 -E 20 -H a	0.984	0.029	0.984	0.984	1	Weighted ave.	98.38	1.6241	0	0	0	#	|	d	=	##
Scheme:	weka.classifiers.functions.MultilayerPerceptron -L 0.3 -M 0.2 -N 500 -V 0 -S 0 -E 20 -H t	1	0.046	0.985	0.992	1	1			a	b	c	d	<--	classified	as	
Scheme:	weka.classifiers.functions.MultilayerPerceptron -L 0.3 -M 0.2 -N 500 -V 0 -S 0 -E 20 -H t	0.967	0	1	0.983	1	2			##	0	0	0	|	a	=	1
Scheme:	weka.classifiers.functions.MultilayerPerceptron -L 0.3 -M 0.2 -N 500 -V 0 -S 0 -E 20 -H t	0.917	0	1	0.957	1	3			2	58	0	0	|	b	=	2
Scheme:	weka.classifiers.functions.MultilayerPerceptron -L 0.3 -M 0.2 -N 500 -V 0 -S 0 -E 20 -H t	1	0	1	1	1	999			3	0	33	0	|	c	=	3
Scheme:	weka.classifiers.functions.MultilayerPerceptron -L 0.3 -M 0.2 -N 500 -V 0 -S 0 -E 20 -H t	0.988	0.035	0.989	0.988	1	Weighted ave.	98.84	1.1601	0	0	0	#	|	d	=	##
Scheme:	weka.classifiers.functions.SimpleLogistic -I 0 -M 500 -H 50 -W 0.0	0.981	0.565	0.839	0.904	0.6	1			a	b	c	d	<--	classified	as	
Scheme:	weka.classifiers.functions.SimpleLogistic -I 0 -M 500 -H 50 -W 0.0	0.183	0.011	0.733	0.293	0.3	2			##	4	2	0	|	a	=	1
Scheme:	weka.classifiers.functions.SimpleLogistic -I 0 -M 500 -H 50 -W 0.0	0.75	0.013	0.844	0.794	0.8	3			46	11	3	0	|	b	=	2
Scheme:	weka.classifiers.functions.SimpleLogistic -I 0 -M 500 -H 50 -W 0.0	0.5	0	1	0.667	0.7	999			9	0	27	0	|	c	=	3
Scheme:	weka.classifiers.functions.SimpleLogistic -I 0 -M 500 -H 50 -W 0.0	0.838	0.426	0.829	0.804	0.5	Weighted ave.	83.76	16.241	6	0	0	6	|	d	=	##
Scheme:	weka.classifiers.lazy.IBk -K 1 -W 0 -A "weka.core.neighboursearch.LinearNNSearch -A \"weka.core.EuclideanDistance -R first-last\""	1	0	1	1	1	1			a	b	c	d	<--	classified	as	
Scheme:	weka.classifiers.lazy.IBk -K 1 -W 0 -A "weka.core.neighboursearch.LinearNNSearch -A \"weka.core.EuclideanDistance -R first-last\""	1	0	1	1	1	2			##	0	0	0	|	a	=	1
Scheme:	weka.classifiers.lazy.IBk -K 1 -W 0 -A "weka.core.neighboursearch.LinearNNSearch -A \"weka.core.EuclideanDistance -R first-last\""	1	0	1	1	1	3			0	60	0	0	|	b	=	2
Scheme:	weka.classifiers.lazy.IBk -K 1 -W 0 -A "weka.core.neighboursearch.LinearNNSearch -A \"weka.core.EuclideanDistance -R first-last\""	1	0	1	1	1	999			0	0	36	0	|	c	=	3
Scheme:	weka.classifiers.lazy.IBk -K 1 -W 0 -A "weka.core.neighboursearch.LinearNNSearch -A \"weka.core.EuclideanDistance -R first-last\""	1	0	1	1	1	Weighted ave.	100	0	0	0	0	#	|	d	=	##
Scheme:	weka.classifiers.lazy.KStar -B 20 -M a	1	0	1	1	1	1			a	b	c	d	<--	classified	as	
Scheme:	weka.classifiers.lazy.KStar -B 20 -M a	1	0	1	1	1	2			##	0	0	0	|	a	=	1
Scheme:	weka.classifiers.lazy.KStar -B 20 -M a	1	0	1	1	1	3			0	60	0	0	|	b	=	2
Scheme:	weka.classifiers.lazy.KStar -B 20 -M a	1	0	1	1	1	999			0	0	36	0	|	c	=	3
Scheme:	weka.classifiers.lazy.KStar -B 20 -M a	1	0	1	1	1	Weighted ave.	100	0	0	0	0	#	|	d	=	##
Scheme:	weka.classifiers.lazy.LWL -U 0 -K -1 -A "weka.core.neighboursearch.LinearNNSearch -A \"weka.core.EuclideanDistance -R first-last\"" -W weka.classifiers.trees.DecisionStump	0.972	0.685	0.809	0.883	0.4	1			a	b	c	d	<--	classified	as	
Scheme:	weka.classifiers.lazy.LWL -U 0 -K -1 -A "weka.core.neighboursearch.LinearNNSearch -A \"weka.core.EuclideanDistance -R first-last\"" -W weka.classifiers.trees.DecisionStump	0	0	?	?	?	2			##	0	9	0	|	a	=	1
Scheme:	weka.classifiers.lazy.LWL -U 0 -K -1 -A "weka.core.neighboursearch.LinearNNSearch -A \"weka.core.EuclideanDistance -R first-last\"" -W weka.classifiers.trees.DecisionStump	0.528	0.03	0.613	0.567	0.5	3			57	0	3	0	|	b	=	2
Scheme:	weka.classifiers.lazy.LWL -U 0 -K -1 -A "weka.core.neighboursearch.LinearNNSearch -A \"weka.core.EuclideanDistance -R first-last\"" -W weka.classifiers.trees.DecisionStump	1	0	1	1	1	999			17	0	19	0	|	c	=	3
Scheme:	weka.classifiers.lazy.LWL -U 0 -K -1 -A "weka.core.neighboursearch.LinearNNSearch -A \"weka.core.EuclideanDistance -R first-last\"" -W weka.classifiers.trees.DecisionStump	0.8	0.516	?	?	?	Weighted ave.	80.05	19.954	0	0	0	#	|	d	=	##
Scheme:	weka.classifiers.meta.AdaBoostM1 -P 100 -S 1 -I 10 -W weka.classifiers.trees.DecisionStump	1	0.889	0.771	0.871	0.3	1			a	b	c	d	<--	classified	as	
Scheme:	weka.classifiers.meta.AdaBoostM1 -P 100 -S 1 -I 10 -W weka.classifiers.trees.DecisionStump	0	0	?	?	?	2			##	0	0	0	|	a	=	1
Scheme:	weka.classifiers.meta.AdaBoostM1 -P 100 -S 1 -I 10 -W weka.classifiers.trees.DecisionStump	0	0	?	?	?	3			60	0	0	0	|	b	=	2
Scheme:	weka.classifiers.meta.AdaBoostM1 -P 100 -S 1 -I 10 -W weka.classifiers.trees.DecisionStump	1	0	1	1	1	999			36	0	0	0	|	c	=	3
Scheme:	weka.classifiers.meta.AdaBoostM1 -P 100 -S 1 -I 10 -W weka.classifiers.trees.DecisionStump	0.777	0.666	?	?	?	Weighted ave.	77.73	22.274	0	0	0	#	|	d	=	##
Scheme:	weka.classifiers.meta.AdaBoostM1 -P 100 -S 1 -I 10 -W weka.classifiers.trees.RandomForest -- -P 100 -I 100 -num-slots 1 -K 0 -M 1.0 -V 0.001 -S 1	1	0	1	1	1	1			a	b	c	d	<--	classified	as	
Scheme:	weka.classifiers.meta.AdaBoostM1 -P 100 -S 1 -I 10 -W weka.classifiers.trees.RandomForest -- -P 100 -I 100 -num-slots 1 -K 0 -M 1.0 -V 0.001 -S 1	1	0	1	1	1	2			##	0	0	0	|	a	=	1
Scheme:	weka.classifiers.meta.AdaBoostM1 -P 100 -S 1 -I 10 -W weka.classifiers.trees.RandomForest -- -P 100 -I 100 -num-slots 1 -K 0 -M 1.0 -V 0.001 -S 1	1	0	1	1	1	3			0	60	0	0	|	b	=	2
Scheme:	weka.classifiers.meta.AdaBoostM1 -P 100 -S 1 -I 10 -W weka.classifiers.trees.RandomForest -- -P 100 -I 100 -num-slots 1 -K 0 -M 1.0 -V 0.001 -S 1	1	0	1	1	1	999			0	0	36	0	|	c	=	3
Scheme:	weka.classifiers.meta.AdaBoostM1 -P 100 -S 1 -I 10 -W weka.classifiers.trees.RandomForest -- -P 100 -I 100 -num-slots 1 -K 0 -M 1.0 -V 0.001 -S 1	1	0	1	1	1	Weighted ave.	100	0	0	0	0	#	|	d	=	##
Scheme:	weka.classifiers.meta.AttributeSelectedClassifier -E "weka.attributeSelection.CfsSubsetEval -P 1 -E 1" -S "weka.attributeSelection.BestFirst -D 1 -N 5" -W weka.classifiers.trees.J48 -- -C 0.25 -M 2	0.997	0.009	0.997	0.997	1	1			a	b	c	d	<--	classified	as	
Scheme:	weka.classifiers.meta.AttributeSelectedClassifier -E "weka.attributeSelection.CfsSubsetEval -P 1 -E 1" -S "weka.attributeSelection.BestFirst -D 1 -N 5" -W weka.classifiers.trees.J48 -- -C 0.25 -M 2	0.967	0	1	0.983	1	2			##	0	1	0	|	a	=	1
Scheme:	weka.classifiers.meta.AttributeSelectedClassifier -E "weka.attributeSelection.CfsSubsetEval -P 1 -E 1" -S "weka.attributeSelection.BestFirst -D 1 -N 5" -W weka.classifiers.trees.J48 -- -C 0.25 -M 2	0.972	0.008	0.921	0.946	0.9	3			0	58	2	0	|	b	=	2
Scheme:	weka.classifiers.meta.AttributeSelectedClassifier -E "weka.attributeSelection.CfsSubsetEval -P 1 -E 1" -S "weka.attributeSelection.BestFirst -D 1 -N 5" -W weka.classifiers.trees.J48 -- -C 0.25 -M 2	1	0	1	1	1	999			1	0	35	0	|	c	=	3
Scheme:	weka.classifiers.meta.AttributeSelectedClassifier -E "weka.attributeSelection.CfsSubsetEval -P 1 -E 1" -S "weka.attributeSelection.BestFirst -D 1 -N 5" -W weka.classifiers.trees.J48 -- -C 0.25 -M 2	0.991	0.008	0.991	0.991	1	Weighted ave.	99.07	0.9281	0	0	0	#	|	d	=	##
Scheme:	weka.classifiers.meta.RandomizableFilteredClassifier -F "weka.filters.unsupervised.attribute.RandomProjection -N 10 -R 42 -D Sparse1" -S 1 -W weka.classifiers.lazy.IBk -- -K 1 -W 0 -A "weka.core.neighboursearch.LinearNNSearch -A \"weka.core.EuclideanDistance -R first-last\""	0.994	0	1	0.997	1	1			a	b	c	d	<--	classified	as	
Scheme:	weka.classifiers.meta.RandomizableFilteredClassifier -F "weka.filters.unsupervised.attribute.RandomProjection -N 10 -R 42 -D Sparse1" -S 1 -W weka.classifiers.lazy.IBk -- -K 1 -W 0 -A "weka.core.neighboursearch.LinearNNSearch -A \"weka.core.EuclideanDistance -R first-last\""	1	0	1	1	1	2			##	0	2	0	|	a	=	1
Scheme:	weka.classifiers.meta.RandomizableFilteredClassifier -F "weka.filters.unsupervised.attribute.RandomProjection -N 10 -R 42 -D Sparse1" -S 1 -W weka.classifiers.lazy.IBk -- -K 1 -W 0 -A "weka.core.neighboursearch.LinearNNSearch -A \"weka.core.EuclideanDistance -R first-last\""	1	0.005	0.947	0.973	1	3			0	60	0	0	|	b	=	2
Scheme:	weka.classifiers.meta.RandomizableFilteredClassifier -F "weka.filters.unsupervised.attribute.RandomProjection -N 10 -R 42 -D Sparse1" -S 1 -W weka.classifiers.lazy.IBk -- -K 1 -W 0 -A "weka.core.neighboursearch.LinearNNSearch -A \"weka.core.EuclideanDistance -R first-last\""	1	0	1	1	1	999			0	0	36	0	|	c	=	3
Scheme:	weka.classifiers.meta.RandomizableFilteredClassifier -F "weka.filters.unsupervised.attribute.RandomProjection -N 10 -R 42 -D Sparse1" -S 1 -W weka.classifiers.lazy.IBk -- -K 1 -W 0 -A "weka.core.neighboursearch.LinearNNSearch -A \"weka.core.EuclideanDistance -R first-last\""	0.995	0	0.996	0.995	1	Weighted ave.	99.54	0.464	0	0	0	#	|	d	=	##
Scheme:	weka.classifiers.meta.LogitBoost -P 100 -L -1.7976931348623157E308 -H 1.0 -Z 3.0 -O 1 -E 1 -S 1 -I 10 -W weka.classifiers.trees.DecisionStump	0.997	0.046	0.985	0.991	1	1			a	b	c	d	<--	classified	as	
Scheme:	weka.classifiers.meta.LogitBoost -P 100 -L -1.7976931348623157E308 -H 1.0 -Z 3.0 -O 1 -E 1 -S 1 -I 10 -W weka.classifiers.trees.DecisionStump	0.883	0.003	0.981	0.93	0.9	2			##	1	0	0	|	a	=	1
Scheme:	weka.classifiers.meta.LogitBoost -P 100 -L -1.7976931348623157E308 -H 1.0 -Z 3.0 -O 1 -E 1 -S 1 -I 10 -W weka.classifiers.trees.DecisionStump	1	0.005	0.947	0.973	1	3			5	53	2	0	|	b	=	2
Scheme:	weka.classifiers.meta.LogitBoost -P 100 -L -1.7976931348623157E308 -H 1.0 -Z 3.0 -O 1 -E 1 -S 1 -I 10 -W weka.classifiers.trees.DecisionStump	1	0	1	1	1	999			0	0	36	0	|	c	=	3
Scheme:	weka.classifiers.meta.LogitBoost -P 100 -L -1.7976931348623157E308 -H 1.0 -Z 3.0 -O 1 -E 1 -S 1 -I 10 -W weka.classifiers.trees.DecisionStump	0.981	0.035	0.982	0.981	1	Weighted ave.	98.14	1.8561	0	0	0	#	|	d	=	##
Scheme:	weka.classifiers.meta.LogitBoost -P 100 -L -1.7976931348623157E308 -H 1.0 -Z 3.0 -O 1 -E 1 -S 1 -I 10 -W weka.classifiers.trees.RandomForest -- -P 100 -I 100 -num-slots 1 -K 0 -M 1.0 -V 0.001 -S 1	1	0	1	1	1	1			a	b	c	d	<--	classified	as	
Scheme:	weka.classifiers.meta.LogitBoost -P 100 -L -1.7976931348623157E308 -H 1.0 -Z 3.0 -O 1 -E 1 -S 1 -I 10 -W weka.classifiers.trees.RandomForest -- -P 100 -I 100 -num-slots 1 -K 0 -M 1.0 -V 0.001 -S 1	1	0	1	1	1	2			##	0	0	0	|	a	=	1
Scheme:	weka.classifiers.meta.LogitBoost -P 100 -L -1.7976931348623157E308 -H 1.0 -Z 3.0 -O 1 -E 1 -S 1 -I 10 -W weka.classifiers.trees.RandomForest -- -P 100 -I 100 -num-slots 1 -K 0 -M 1.0 -V 0.001 -S 1	1	0	1	1	1	3			0	60	0	0	|	b	=	2
Scheme:	weka.classifiers.meta.LogitBoost -P 100 -L -1.7976931348623157E308 -H 1.0 -Z 3.0 -O 1 -E 1 -S 1 -I 10 -W weka.classifiers.trees.RandomForest -- -P 100 -I 100 -num-slots 1 -K 0 -M 1.0 -V 0.001 -S 1	1	0	1	1	1	999			0	0	36	0	|	c	=	3
Scheme:	weka.classifiers.meta.LogitBoost -P 100 -L -1.7976931348623157E308 -H 1.0 -Z 3.0 -O 1 -E 1 -S 1 -I 10 -W weka.classifiers.trees.RandomForest -- -P 100 -I 100 -num-slots 1 -K 0 -M 1.0 -V 0.001 -S 1	1	0	1	1	1	Weighted ave.	100	0	0	0	0	#	|	d	=	##

Feature selection OD:

Evaluator: weka.attributeSelection.CfsSubsetEval -P 1 -E 1
Search: weka.attributeSelection.GreedyStepwise -T -1.7976931348623157E308 -N -1 -num-slots 1

Age
OSDI
OSSOD
Cornsens OS
MEOS
MOOS
OsmOS
ST1OS
OSSOS

Evaluator: weka.attributeSelection.ClassifierAttributeEval -execution-slots 1 -B weka.classifiers.rules.ZeroR -F 5 -T 0.01 -R 1 -E DEFAULT --
Search: weka.attributeSelection.Ranker -T -1.7976931348623157E308 -N -1

0 25 OSSOS
0 24 ST1OS
0 8 OSDI
0 9 Blinkfreq OD

0	10 Cornsens OD
0	7 Age80-99
0	6 Age60-79
0	5 Age40-59
0	2 Sex
0	3 Age0-19
0	4 Age20-39
0	12 MeiboOD
0	13 MEOD
0	14 MOOD
0	22 MOOS
0	23 OsmOS
0	20 MeiboOS
0	21 MEOS
0	19 Cornsens OS
0	15 OsmOD
0	16 ST1OD
0	17 OSSOD
0	18 Blinkfreq OS
0	1 Age

Evaluator: weka.attributeSelection.ClassifierSubsetEval -B weka.classifiers.trees.RandomForest -T -H "Click to set hold out or test instances" -E DEFAULT -- -P 100 -I 100 -num-slots 1 -K 0 -M 1.0 -V 0.001 -S 1
Search: weka.attributeSelection.BestFirst -D 1 -N 5

Age

Evaluator: weka.attributeSelection.CorrelationAttributeEval
Search: weka.attributeSelection.Ranker -T -1.7976931348623157E308 -N -1

0.315	8 OSDI
0.2875	24 ST1OS
0.2822	17 OSSOD
0.2815	25 OSSOS
0.2187	16 ST1OD
0.218	6 Age60-79
0.2166	5 Age40-59
0.2146	13 MEOD
0.2144	21 MEOS
0.2098	23 OsmOS
0.1841	9 Blinkfreq OD
0.1841	18 Blinkfreq OS
0.1603	15 OsmOD
0.1383	2 Sex
0.1321	19 Cornsens OS
0.1306	10 Cornsens OD
0.1016	22 MOOS
0.1006	14 MOOD
0.0976	12 MeiboOD
0.0974	20 MeiboOS
0.0691	7 Age80-99
0.0534	3 Age0-19
0.0494	4 Age20-39
0.0434	1 Age

Evaluator: weka.attributeSelection.GainRatioAttributeEval
Search: weka.attributeSelection.Ranker -T -1.7976931348623157E308 -N -1

0.2638	1 Age
0.2245	17 OSSOD
0.2178	24 ST1OS
0.1797	25 OSSOS
0.1752	8 OSDI
0.1696	23 OsmOS
0.1623	16 ST1OD
0.1606	21 MEOS
0.1579	22 MOOS
0.1499	13 MEOD
0.1498	19 Cornsens OS
0.1436	15 OsmOD
0.1224	9 Blinkfreq OD
0.1224	18 Blinkfreq OS
0.109	10 Cornsens OD
0.1065	14 MOOD
0.0931	20 MeiboOS
0.077	12 MeiboOD
0.0691	5 Age40-59
0.0491	2 Sex
0.0429	6 Age60-79
0	3 Age0-19
0	4 Age20-39
0	7 Age80-99

Evaluator: weka.attributeSelection.InfoGainAttributeEval
Search: weka.attributeSelection.Ranker -T -1.7976931348623157E308 -N -1

0.8643	1 Age
0.7629	24 ST1OS
0.4803	8 OSDI
0.4622	16 ST1OD
0.3479	14 MOOD
0.3209	22 MOOS
0.2897	17 OSSOD
0.2885	25 OSSOS
0.2658	18 Blinkfreq OS
0.2658	9 Blinkfreq OD
0.2273	23 OsmOS
0.2129	21 MEOS
0.2044	13 MEOD
0.2031	15 OsmOD
0.1717	10 Cornsens OD
0.1287	12 MeiboOD
0.1121	19 Cornsens OS
0.0976	20 MeiboOS
0.0654	5 Age40-59
0.0399	6 Age60-79
0.0386	2 Sex
0	3 Age0-19
0	4 Age20-39
0	7 Age80-99

Evaluator: weka.attributeSelection.OneRAttributeEval -S 1 -F 10 -B 6
Search: weka.attributeSelection.Ranker -T -1.7976931348623157E308 -N -1

92.34339	1 Age
87.00696	24 ST1OS
80.27842	17 OSSOD
80.27842	25 OSSOS
79.81439	16 ST1OD
79.58237	15 OsmOD
79.58237	23 OsmOS
79.11833	21 MEOS
79.11833	13 MEOD
78.88631	8 OSDI
78.19026	10 Cornsens OD
77.95824	22 MOOS
77.4942	6 Age60-79
77.4942	4 Age20-39
77.4942	3 Age0-19
77.4942	2 Sex
77.4942	5 Age40-59
77.4942	7 Age80-99
77.4942	20 MeiboOS
77.4942	12 MeiboOD
76.56613	14 MOOD
76.33411	19 Cornsens OS
76.10209	18 Blinkfreq OS
76.10209	9 Blinkfreq OD

Evaluator: weka.attributeSelection.PrincipalComponents -R 0.95 -A 5
Search: weka.attributeSelection.Ranker -T -1.7976931348623157E308 -N -1

0.6788	1 -0.327ST1OD-0.327MEOS-0.326MEOD-0.309OSSOS-0.308OSSOD...
0.543	2 -0.438Blinkfreq OD-0.438Blinkfreq OS-0.433Cornsens OD-0.433Cornsens OS+0.173MeiboOS...
0.4401	3 -0.458Age60-79-0.448Age+0.376OsmOS+0.305OsmOD+0.282Age20-39...
0.3567	4 -0.48OsmOD+0.468Age20-39-0.411OsmOS-0.394Age-0.253Age40-59...
0.2862	5 0.485Age40-59+0.479OSDI+0.337MOOD+0.336MOOS-0.29Age60-79...
0.2331	6 0.377OSDI-0.316Age80-99-0.312Age40-59+0.275Age20-39+0.273OsmOS...
0.1855	7 0.659Age80-99-0.36Sex-0.336Age40-59+0.239Age20-39+0.226MOOS...
0.1419	8 0.49 MeiboOD+0.49 MeiboOS-0.358Sex-0.262MOOS-0.259MOOD...
0.0991	9 0.843Age0-19-0.299Age80-99-0.255Sex+0.201Age60-79-0.162Age40-59...
0.0637	10 0.66 Sex+0.349Age80-99+0.343Age0-19-0.206ST1OS-0.195Age20-39...
0.0371	11 0.635OSDI+0.333Sex+0.279ST1OS+0.278OSSOS+0.278OSSOD...

Evaluator: weka.attributeSelection.ReliefFAttributeEval -M -1 -D 1 -K 10
Search: weka.attributeSelection.Ranker -T -1.7976931348623157E308 -N -1

0.23629	23 OsmOS
0.20423	2 Sex
0.19852	8 OSDI
0.19696	15 OsmOD
0.17065	5 Age40-59
0.16933	10 Cornsens OD
0.16494	19 Cornsens OS
0.13355	24 ST1OS
0.13062	25 OSSOS
0.13044	17 OSSOD
0.12489	1 Age
0.12284	4 Age20-39
0.12167	9 Blinkfreq OD
0.12167	18 Blinkfreq OS
0.10189	6 Age60-79
0.0724	20 MeiboOS
0.07237	12 MeiboOD
0.06715	22 MOOS
0.06694	14 MOOD
0.0586	16 ST1OD
0.05425	21 MEOS
0.05422	13 MEOD
0.02485	7 Age80-99
0.00325	3 Age0-19

Evaluator: weka.attributeSelection.SymmetricalUncertAttributeEval
Search: weka.attributeSelection.Ranker -T -1.7976931348623157E308 -N -1

0.3986	1 Age
0.3344	24 ST1OS
0.2527	8 OSDI
0.2465	17 OSSOD
0.2366	16 ST1OD
0.2165	25 OSSOS
0.2075	22 MOOS
0.1894	23 OsmOS
0.1785	21 MEOS
0.1687	13 MEOD
0.1645	18 Blinkfreq OS
0.1645	9 Blinkfreq OD
0.1641	15 OsmOD
0.1608	14 MOOD
0.1303	10 Cornsens OD

0.124	19	Cornsens OS
0.0942	12	MeiboOD
0.0926	20	MeiboOS
0.0652	5	Age40-59
0.0418	2	Sex
0.0401	6	Age60-79
0	3	Age0-19
0	7	Age80-99
0	4	Age20-39

Evaluator: weka.attributeSelection.WrapperSubsetEval -B weka.classifiers.trees.RandomForest -F 5 -T 0.01 -R 1 -E DEFAULT -- -P 100 -I 100 -num-slots 1 -K 0 -M 1.0 -V 0.001 -S 1
Search: weka.attributeSelection.GreedyStepwise -T -1.7976931348623157E308 -N -1 -num-slots 1

Age
Age80-99
OSDI

Evaluator: weka.attributeSelection.ClassifierAttributeEval -execution-slots 1 -B weka.classifiers.trees.RandomForest -F 5 -T 0.01 -R 1 -E DEFAULT -- -P 100 -I 100 -num-slots 1 -K 0 -M 1.0 -V 0.001 -S 1
Search: weka.attributeSelection.Ranker -T -1.7976931348623157E308 -N -1

0.2181	1	Age
0.11601	24	ST1OS
0.06265	16	ST1OD
0.04733	8	OSDI
0.02784	17	OSSOD
0.02784	15	OsmOD
0.02784	25	OSSOS
0.02784	23	OsmOS
0.01624	13	MEOD
0.01624	21	MEOS
0.01253	10	Cornsens OD
0.00139	22	MOOS
0	2	Sex
0	6	Age60-79
0	5	Age40-59
0	7	Age80-99
0	20	MeiboOS
0	4	Age20-39
0	3	Age0-19
0	12	MeiboOD
-0.00928	19	Cornsens OS
-0.00974	18	Blinkfreq OS
-0.00974	9	Blinkfreq OD
-0.01624	14	MOOD

Evaluator: weka.attributeSelection.ClassifierAttributeEval -execution-slots 1 -B weka.classifiers.meta.RandomizableFilteredClassifier -F 5 -T 0.01 -R 1 -E DEFAULT -- -F "weka.filters.unsupervised.attribute.RandomProjection -N 10 -R 42 -D Sparse1" -S 1 -W weka.classifiers.lazy.IBk -- -K 1 -W 0 -A "weka.core.
Search: weka.attributeSelection.Ranker -T -1.7976931348623157E308 -N -1

0.2181	1	Age
0.11833	24	ST1OS
0.05916	16	ST1OD
0.04408	8	OSDI
0.02784	17	OSSOD
0.02784	15	OsmOD
0.02784	25	OSSOS
0.02784	23	OsmOS
0.01624	13	MEOD
0.01624	21	MEOS
0.01508	10	Cornsens OD
0.00928	22	MOOS
0	2	Sex
0	6	Age60-79
0	5	Age40-59
0	7	Age80-99
0	20	MeiboOS
0	4	Age20-39
0	3	Age0-19
0	12	MeiboOD
-0.00696	19	Cornsens OS
-0.0116	18	Blinkfreq OS
-0.0116	9	Blinkfreq OD
-0.01253	14	MOOD

Feature selection OS:

Evaluator: weka.attributeSelection.CfsSubsetEval -P 1 -E 1
Search: weka.attributeSelection.BestFirst -D 1 -N 5

Age
OSDI
OsmOS
ST1OS
OSSOS

Evaluator: weka.attributeSelection.CfsSubsetEval -P 1 -E 1
Search: weka.attributeSelection.GreedyStepwise -T -1.7976931348623157E308 -N -1 -num-slots 1

Age
ST1OS

Evaluator: weka.attributeSelection.ClassifierAttributeEval -execution-slots 1 -B weka.classifiers.rules.ZeroR -F 5 -T 0.01 -R 1 -E DEFAULT --
Search: weka.attributeSelection.Ranker -T -1.7976931348623157E308 -N -1

0	25	OSSOS
0	24	ST1OS
0	8	OSDI
0	9	Blinkfreq OD
0	10	Cornsens OD
0	7	Age80-99
0	6	Age60-79
0	5	Age40-59
0	2	Sex
0	3	Age0-19
0	4	Age20-39
0	11	MeiboOD
0	12	MEOD
0	13	MOOD
0	22	MOOS
0	23	OsmOS
0	20	MeiboOS
0	21	MEOS
0	18	Cornsens OS
0	14	OsmOD
0	15	ST1OD
0	16	OSSOD
0	17	Blinkfreq OS
0	1	Age

Evaluator: weka.attributeSelection.ClassifierAttributeEval -execution-slots 1 -B weka.classifiers.trees.RandomForest -F 5 -T 0.01 -R 1 -E DEFAULT -- -P 100 -I 100 -num-slots 1 -K 0 -M 1.0 -V 0.001 -S 1
Search: weka.attributeSelection.Ranker -T -1.7976931348623157E308 -N -1

0.24826	1	Age
0.12181	24	ST1OS
0.11601	15	ST1OD
0.058	8	OSDI
0.04408	9	Blinkfreq OD
0.04408	17	Blinkfreq OS
0.04176	23	OsmOS
0.02784	16	OSSOD
0.02784	25	OSSOS
0.0232	14	OsmOD
0.01624	12	MEOD
0.01624	21	MEOS
0.01624	10	Cornsens OD
0.00696	13	MOOD
0.00348	18	Cornsens OS
0	11	MeiboOD
0	4	Age20-39
0	2	Sex
0	3	Age0-19
0	5	Age40-59
0	20	MeiboOS
0	6	Age60-79
0	7	Age80-99
-0.01671	22	MOOS

Evaluator: weka.attributeSelection.CorrelationAttributeEval
Search: weka.attributeSelection.Ranker -T -1.7976931348623157E308 -N -1

0.2616	24	ST1OS
0.2604	16	OSSOD
0.2602	25	OSSOS
0.1972	12	MEOD
0.1969	21	MEOS
0.1944	15	ST1OD
0.1683	23	OsmOS
0.1554	17	Blinkfreq OS
0.1554	9	Blinkfreq OD
0.1518	10	Cornsens OD
0.151	18	Cornsens OS
0.1384	14	OsmOD
0.1134	5	Age40-59
0.102	7	Age80-99
0.0965	6	Age60-79
0.0903	8	OSDI
0.0892	22	MOOS
0.0888	13	MOOD
0.0863	11	MeiboOD
0.0863	20	MeiboOS
0.0857	4	Age20-39
0.0827	1	Age
0.0563	3	Age0-19
0.0102	2	Sex

Evaluator: weka.attributeSelection.GainRatioAttributeEval
Search: weka.attributeSelection.Ranker -T -1.7976931348623157E308 -N -1

0.2877	1	Age
0.2635	20	MeiboOS
0.2635	11	MeiboOD
0.2263	24	ST1OS
0.2156	25	OSSOS
0.1908	15	ST1OD
0.1904	23	OsmOS
0.1828	8	OSDI
0.169	21	MEOS
0.1671	14	OsmOD
0.1457	17	Blinkfreq OS
0.1457	9	Blinkfreq OD
0.1448	13	MOOD
0.1427	16	OSSOD
0.1391	12	MEOD
0.1309	18	Cornsens OS
0.1224	22	MOOS
0.116	10	Cornsens OD
0.069	7	Age80-99
0.0443	4	Age20-39
0.0308	5	Age40-59
0	2	Sex
0	6	Age60-79
0	3	Age0-19

Evaluator: weka.attributeSelection.InfoGainAttributeEval
Search: weka.attributeSelection.Ranker -T -1.7976931348623157E308 -N -1

1.0536	1	Age
0.8077	24	ST1OS
0.7473	15	ST1OD
0.5113	8	OSDI
0.423	9	Blinkfreq OD
0.423	17	Blinkfreq OS
0.38	22	MOOS
0.3642	13	MOOD
0.3599	16	OSSOD
0.264	23	OsmOS
0.2429	12	MEOD
0.2419	25	OSSOS
0.2241	21	MEOS
0.2131	18	Cornsens OS
0.1801	10	Cornsens OD
0.1207	14	OsmOD
0.0632	20	MeiboOS
0.0632	11	MeiboOD
0.0353	4	Age20-39
0.0291	5	Age40-59
0.0158	7	Age80-99
0	6	Age60-79
0	2	Sex
0	3	Age0-19

Evaluator: weka.attributeSelection.OneRAttributeEval -S 1 -F 10 -B 6
Search: weka.attributeSelection.Ranker -T -1.7976931348623157E308 -N -1

93.271	1	Age
84.919	15	ST1OD
82.831	24	ST1OS
77.726	17	Blinkfreq OS
77.726	9	Blinkfreq OD
77.726	16	OSSOD
77.726	25	OSSOS
77.726	23	OsmOS
76.566	21	MEOS
76.566	12	MEOD
76.102	14	OsmOD
76.102	8	OSDI
74.942	2	Sex
74.942	5	Age40-59
74.942	4	Age20-39
74.942	6	Age60-79
74.942	3	Age0-19
74.942	7	Age80-99
74.942	18	Cornsens OS
74.942	20	MeiboOS
74.942	11	MeiboOD
74.71	10	Cornsens OD
72.854	13	MOOD
71.462	22	MOOS

Evaluator: weka.attributeSelection.PrincipalComponents -R 0.95 -A 5
Search: weka.attributeSelection.Ranker -T -1.7976931348623157E308 -N -1

0.6788	1	-0.327ST1OD-0.327MEOS-0.326MEOD-0.309OSSOS-0.308OSSOD...
0.543	2	-0.438Blinkfreq OD-0.438Blinkfreq OS-0.433Cornsens OD-0.433Cornsens OS+0.173MeiboOS...
0.4401	3	-0.458Age60-79-0.448Age+0.376OsmOS+0.305OsmOD+0.282Age20-39...
0.3567	4	-0.48OsmOD+0.468Age20-39-0.411OsmOS-0.394Age-0.253Age40-59...
0.2862	5	0.485Age40-59+0.479OSDI+0.337MOOD+0.336MOOS-0.29Age60-79...
0.2331	6	0.377OSDI-0.316Age80-99-0.312Age40-59+0.275Age20-39+0.273OsmOS...
0.1855	7	0.659Age80-99-0.36Sex-0.336Age40-59+0.239Age20-39+0.226MOOS...
0.1419	8	0.49 MeiboOD+0.49 MeiboOS-0.358Sex-0.262MOOS-0.259MOOD...
0.0991	9	0.843Age0-19-0.299Age80-99-0.255Sex+0.201Age60-79-0.162Age40-59...
0.0637	10	0.66 Sex+0.349Age80-99+0.343Age0-19-0.206ST1OS-0.195Age20-39...
0.0371	11	0.635OSDI+0.333Sex+0.279ST1OS+0.278OSSOS+0.278OSSOD...

Evaluator: weka.attributeSelection.ReliefFAttributeEval -M -1 -D 1 -K 10
Search: weka.attributeSelection.Ranker -T -1.7976931348623157E308 -N -1

0.21319	23	OsmOS
0.19101	8	OSDI
0.17739	14	OsmOD
0.16499	2	Sex
0.16188	5	Age40-59
0.12193	24	ST1OS
0.11915	25	OSSOS
0.1188	16	OSSOD
0.11831	10	Cornsens OD
0.11667	18	Cornsens OS
0.10693	1	Age
0.09442	4	Age20-39
0.09118	17	Blinkfreq OS
0.09118	9	Blinkfreq OD
0.09094	6	Age60-79
0.06688	11	MeiboOD
0.06684	20	MeiboOS
0.05888	22	MOOS
0.0588	13	MOOD
0.05225	15	ST1OD
0.04845	21	MEOS
0.04835	12	MEOD
0.02552	7	Age80-99
0.00325	3	Age0-19

Evaluator: weka.attributeSelection.SymmetricalUncertAttributeEval
Search: weka.attributeSelection.Ranker -T -1.7976931348623157E308 -N -1

0.4378	1	Age
0.3423	24	ST1OS
0.2949	15	ST1OD
0.259	8	OSDI
0.2128	25	OSSOS
0.2086	9	Blinkfreq OD
0.2086	17	Blinkfreq OS
0.2081	23	OsmOS
0.1987	13	MOOD
0.196	16	OSSOD
0.181	21	MEOS
0.1785	22	MOOS
0.1677	12	MEOD
0.1534	18	Cornsens OS
0.1333	10	Cornsens OD
0.1289	14	OsmOD
0.0909	11	MeiboOD
0.0909	20	MeiboOS
0.0363	4	Age20-39
0.0278	5	Age40-59
0.0229	7	Age80-99
0	2	Sex
0	6	Age60-79
0	3	Age0-19

GridSearch OD

Scheme	Recall	FP Rate	Precision	F-Measure	MCC	Class	Correct	Incorrect								
Scheme: RandomForest: weka.classifiers.meta.GridSearch -E CC -v-property numIterations -v-min -3.0 -v-max 3.0 -v-step 1.0 -v-base 10.0 -v-expression pow(BASE,I) -x-property maxDepth -x-min -3.0 -x-max 3.0 -x-step 1.0 -x-base 10.0 -x-expression pow(BASE,I) -sample-size 100.0 -traversal ROW-WISE	1	0.109	0.971	0.985	0.9	1			a	b	c	d	<--	classified	as	
Scheme: weka.classifiers.meta.GridSearch -E CC -v-property numIterations -v-min -3.0 -v-max 3.0 -v-step 1.0 -v-base 10.0 -v-expression pow(BASE,I) -x-property maxDepth -x-min -3.0 -x-max 3.0 -x-step 1.0 -x-base 10.0 -x-expression pow(BASE,I) -sample-size 100.0 -traversal ROW-WISE -log-file "C:\\P	0.844	0	1	0.915	0.9	2			##	0	0	0	l	a	=	1
Scheme: weka.classifiers.meta.GridSearch -E CC -v-property numIterations -v-min -3.0 -v-max 3.0 -v-step 1.0 -v-base 10.0 -v-expression pow(BASE,I) -x-property maxDepth -x-min -3.0 -x-max 3.0 -x-step 1.0 -x-base 10.0 -x-expression pow(BASE,I) -sample-size 100.0 -traversal ROW-WISE -log-file "C:\\P	1	0	1	1	1	3			5	27	0	0	l	b	=	2
Scheme: weka.classifiers.meta.GridSearch -E CC -v-property numIterations -v-min -3.0 -v-max 3.0 -v-step 1.0 -v-base 10.0 -v-expression pow(BASE,I) -x-property maxDepth -x-min -3.0 -x-max 3.0 -x-step 1.0 -x-base 10.0 -x-expression pow(BASE,I) -sample-size 100.0 -traversal ROW-WISE -log-file "C:\\P	1	0	1	1	1	999			0	0	12	0	l	c	=	3
Scheme: weka.classifiers.meta.GridSearch -E CC -v-property numIterations -v-min -3.0 -v-max 3.0 -v-step 1.0 -v-base 10.0 -v-expression pow(BASE,I) -x-property maxDepth -x-min -3.0 -x-max 3.0 -x-step 1.0 -x-base 10.0 -x-expression pow(BASE,I) -sample-size 100.0 -traversal ROW-WISE -log-file "C:\\P	0.977	0.085	0.977	0.976	0.9	Weighted Avg.	97.67	2.3256	0	0	0	2	l	d	=	##
Scheme: NaiveBayes weka.classifiers.meta.GridSearch -E CC -v-property useKernelEstimator -v-min -3.0 -v-max 3.0 -v-step 1.0 -v-base 10.0 -v-expression pow(BASE,I) -x-property debug -x-min -3.0 -x-max 3.0 -x-step 1.0 -x-base 10.0 -x-expression pow(BASE,I) -sample-size 100.0 -traversal ROW-WISE -l	1	0.522	0.876	0.934	0.6	1			a	b	c	d	<--	classified	as	
Scheme: weka.classifiers.meta.GridSearch -E CC -v-property useKernelEstimator -v-min -3.0 -v-max 3.0 -v-step 1.0 -v-base 10.0 -v-expression pow(BASE,I) -x-property debug -x-min -3.0 -x-max 3.0 -x-step 1.0 -x-base 10.0 -x-expression pow(BASE,I) -sample-size 100.0 -traversal ROW-WISE -log-file "C:\\I	0.469	0	1	0.638	0.7	2			##	0	0	0	l	a	=	1
Scheme: weka.classifiers.meta.GridSearch -E CC -v-property useKernelEstimator -v-min -3.0 -v-max 3.0 -v-step 1.0 -v-base 10.0 -v-expression pow(BASE,I) -x-property debug -x-min -3.0 -x-max 3.0 -x-step 1.0 -x-base 10.0 -x-expression pow(BASE,I) -sample-size 100.0 -traversal ROW-WISE -log-file "C:\\I	0.417	0	1	0.588	0.6	3			17	15	0	0	l	b	=	2
Scheme: weka.classifiers.meta.GridSearch -E CC -v-property useKernelEstimator -v-min -3.0 -v-max 3.0 -v-step 1.0 -v-base 10.0 -v-expression pow(BASE,I) -x-property debug -x-min -3.0 -x-max 3.0 -x-step 1.0 -x-base 10.0 -x-expression pow(BASE,I) -sample-size 100.0 -traversal ROW-WISE -log-file "C:\\I	1	0	1	1	1	999			7	0	5	0	l	c	=	3
Scheme: weka.classifiers.meta.GridSearch -E CC -v-property useKernelEstimator -v-min -3.0 -v-max 3.0 -v-step 1.0 -v-base 10.0 -v-expression pow(BASE,I) -x-property debug -x-min -3.0 -x-max 3.0 -x-step 1.0 -x-base 10.0 -x-expression pow(BASE,I) -sample-size 100.0 -traversal ROW-WISE -log-file "C:\\I	0.888	0.41	0.902	0.871	0.7	Weighted Avg.	88.84	11.163	0	0	0	2	l	d	=	##
Scheme: RandomizableFilteredClassifier+IBk weka.classifiers.meta.GridSearch -E CC -v-property seed -v-min -3.0 -v-max 3.0 -v-step 1.0 -v-base 10.0 -v-expression pow(BASE,I) -x-property debug -x-min -3.0 -x-max 3.0 -x-step 1.0 -x-base 10.0 -x-expression pow(BASE,I) -sample-size 100.0 -traversal ROW	0.976	0.065	0.982	0.979	0.9	1			a	b	c	d	<--	classified	as	
Scheme: weka.classifiers.meta.GridSearch -E CC -v-property seed -v-min -3.0 -v-max 3.0 -v-step 1.0 -v-base 10.0 -v-expression pow(BASE,I) -x-property debug -x-min -3.0 -x-max 3.0 -x-step 1.0 -x-base 10.0 -x-expression pow(BASE,I) -sample-size 100.0 -traversal ROW-WISE -log-file "C:\\Program Files\\	0.906	0.022	0.879	0.892	0.9	2			##	4	0	0	l	a	=	1
Scheme: weka.classifiers.meta.GridSearch -E CC -v-property seed -v-min -3.0 -v-max 3.0 -v-step 1.0 -v-base 10.0 -v-expression pow(BASE,I) -x-property debug -x-min -3.0 -x-max 3.0 -x-step 1.0 -x-base 10.0 -x-expression pow(BASE,I) -sample-size 100.0 -traversal ROW-WISE -log-file "C:\\Program Files\\	1	0	1	1	1	3			3	29	0	0	l	b	=	2
Scheme: weka.classifiers.meta.GridSearch -E CC -v-property seed -v-min -3.0 -v-max 3.0 -v-step 1.0 -v-base 10.0 -v-expression pow(BASE,I) -x-property debug -x-min -3.0 -x-max 3.0 -x-step 1.0 -x-base 10.0 -x-expression pow(BASE,I) -sample-size 100.0 -traversal ROW-WISE -log-file "C:\\Program Files\\	1	0	1	1	1	999			0	0	12	0	l	c	=	3
Scheme: weka.classifiers.meta.GridSearch -E CC -v-property seed -v-min -3.0 -v-max 3.0 -v-step 1.0 -v-base 10.0 -v-expression pow(BASE,I) -x-property debug -x-min -3.0 -x-max 3.0 -x-step 1.0 -x-base 10.0 -x-expression pow(BASE,I) -sample-size 100.0 -traversal ROW-WISE -log-file "C:\\Program Files\\	0.967	0.055	0.968	0.968	0.9	Weighted Avg.	96.74	3.2558	0	0	0	2	l	d	=	##
Scheme: MultiLayerPerceptron weka.classifiers.meta.GridSearch -E CC -v-property learningRate -v-min -3.0 -v-max 3.0 -v-step 1.0 -v-base 10.0 -v-expression pow(BASE,I) -x-property momentum -x-min -3.0 -x-max 3.0 -x-step 1.0 -x-base 10.0 -x-expression pow(BASE,I) -sample-size 100.0 -traversal ROW	1	0.087	0.977	0.988	0.9	1			a	b	c	d	<--	classified	as	
Scheme: weka.classifiers.meta.GridSearch -E CC -v-property learningRate -v-min -3.0 -v-max 3.0 -v-step 1.0 -v-base 10.0 -v-expression pow(BASE,I) -x-property momentum -x-min -3.0 -x-max 3.0 -x-step 1.0 -x-base 10.0 -x-expression pow(BASE,I) -sample-size 100.0 -traversal ROW-WISE -log-file "C:\\Pr	0.906	0	1	0.951	0.9	2			##	0	0	0	l	a	=	1
Scheme: weka.classifiers.meta.GridSearch -E CC -v-property learningRate -v-min -3.0 -v-max 3.0 -v-step 1.0 -v-base 10.0 -v-expression pow(BASE,I) -x-property momentum -x-min -3.0 -x-max 3.0 -x-step 1.0 -x-base 10.0 -x-expression pow(BASE,I) -sample-size 100.0 -traversal ROW-WISE -log-file "C:\\Pr	0.75	0.01	0.818	0.783	0.8	3			1	29	2	0	l	b	=	2
Scheme: weka.classifiers.meta.GridSearch -E CC -v-property learningRate -v-min -3.0 -v-max 3.0 -v-step 1.0 -v-base 10.0 -v-expression pow(BASE,I) -x-property momentum -x-min -3.0 -x-max 3.0 -x-step 1.0 -x-base 10.0 -x-expression pow(BASE,I) -sample-size 100.0 -traversal ROW-WISE -log-file "C:\\Pr	1	0	1	1	1	999			3	0	9	0	l	c	=	3
Scheme: weka.classifiers.meta.GridSearch -E CC -v-property learningRate -v-min -3.0 -v-max 3.0 -v-step 1.0 -v-base 10.0 -v-expression pow(BASE,I) -x-property momentum -x-min -3.0 -x-max 3.0 -x-step 1.0 -x-base 10.0 -x-expression pow(BASE,I) -sample-size 100.0 -traversal ROW-WISE -log-file "C:\\Pr	0.972	0.069	0.972	0.971	0.9	Weighted Avg.	97.21	2.7907	0	0	0	2	l	d	=	##

GridSearch OS

Scheme	Recall	FP Rate	Precision	F-Measure	MCC	Class	Correct	Incorrect								
Scheme: NaiveBayes weka.classifiers.meta.GridSearch -E CC -v-property useKernelEstimator -v-min -3.0 -v-max 3.0 -v-step 1.0 -v-base 10.0 -v-expression pow(BASE,I) -x-property debug -x-min -3.0 -x-max 3.0 -x-step 1.0 -x-base 10.0 -x-expression pow(BASE,I) -sample-size 100.0 -traversal ROW-WISE -l	1	0.964	0.746	0.855	0.2	1			a	b	c	d	<--	classified	as	
Scheme: weka.classifiers.meta.GridSearch -E CC -v-property useKernelEstimator -v-min -3.0 -v-max 3.0 -v-step 1.0 -v-base 10.0 -v-expression pow(BASE,I) -x-property debug -x-min -3.0 -x-max 3.0 -x-step 1.0 -x-base 10.0 -x-expression pow(BASE,I) -sample-size 100.0 -traversal ROW-WISE -log-file "C:\\I	0	0	?	?	?	2			##	0	0	0	l	a	=	1
Scheme: weka.classifiers.meta.GridSearch -E CC -v-property useKernelEstimator -v-min -3.0 -v-max 3.0 -v-step 1.0 -v-base 10.0 -v-expression pow(BASE,I) -x-property debug -x-min -3.0 -x-max 3.0 -x-step 1.0 -x-base 10.0 -x-expression pow(BASE,I) -sample-size 100.0 -traversal ROW-WISE -log-file "C:\\I	0	0	?	?	?	3			36	0	0	0	l	b	=	2
Scheme: weka.classifiers.meta.GridSearch -E CC -v-property useKernelEstimator -v-min -3.0 -v-max 3.0 -v-step 1.0 -v-base 10.0 -v-expression pow(BASE,I) -x-property debug -x-min -3.0 -x-max 3.0 -x-step 1.0 -x-base 10.0 -x-expression pow(BASE,I) -sample-size 100.0 -traversal ROW-WISE -log-file "C:\\I	1	0	1	1	1	999			18	0	0	0	l	c	=	3
Scheme: weka.classifiers.meta.GridSearch -E CC -v-property useKernelEstimator -v-min -3.0 -v-max 3.0 -v-step 1.0 -v-base 10.0 -v-expression pow(BASE,I) -x-property debug -x-min -3.0 -x-max 3.0 -x-step 1.0 -x-base 10.0 -x-expression pow(BASE,I) -sample-size 100.0 -traversal ROW-WISE -log-file "C:\\I	0.749	0.713	?	?	?	Weighted Avg.	74.88	25.116	0	0	0	2	l	d	=	##
Scheme: MultiLayerPerceptron weka.classifiers.meta.GridSearch -E CC -v-property momentum -v-min -3.0 -v-max 3.0 -v-step 1.0 -v-base 10.0 -v-expression pow(BASE,I) -x-property learningRate -x-min -3.0 -x-max 3.0 -x-step 1.0 -x-base 10.0 -x-expression pow(BASE,I) -sample-size 100.0 -traversal ROW	1	0.089	0.97	0.985	0.9	1			a	b	c	d	<--	classified	as	
Scheme: weka.classifiers.meta.GridSearch -E CC -v-property momentum -v-min -3.0 -v-max 3.0 -v-step 1.0 -v-base 10.0 -v-expression pow(BASE,I) -x-property learningRate -x-min -3.0 -x-max 3.0 -x-step 1.0 -x-base 10.0 -x-expression pow(BASE,I) -sample-size 100.0 -traversal ROW-WISE -log-file "C:\\Pr	0.833	0	1	0.909	0.9	2			##	0	0	0	l	a	=	1
Scheme: weka.classifiers.meta.GridSearch -E CC -v-property momentum -v-min -3.0 -v-max 3.0 -v-step 1.0 -v-base 10.0 -v-expression pow(BASE,I) -x-property learningRate -x-min -3.0 -x-max 3.0 -x-step 1.0 -x-base 10.0 -x-expression pow(BASE,I) -sample-size 100.0 -traversal ROW-WISE -log-file "C:\\Pr	0.889	0.015	0.842	0.865	0.9	3			3	30	3	0	l	b	=	2
Scheme: weka.classifiers.meta.GridSearch -E CC -v-property momentum -v-min -3.0 -v-max 3.0 -v-step 1.0 -v-base 10.0 -v-expression pow(BASE,I) -x-property learningRate -x-min -3.0 -x-max 3.0 -x-step 1.0 -x-base 10.0 -x-expression pow(BASE,I) -sample-size 100.0 -traversal ROW-WISE -log-file "C:\\Pr	1	0	1	1	1	999			2	0	16	0	l	c	=	3
Scheme: weka.classifiers.meta.GridSearch -E CC -v-property momentum -v-min -3.0 -v-max 3.0 -v-step 1.0 -v-base 10.0 -v-expression pow(BASE,I) -x-property learningRate -x-min -3.0 -x-max 3.0 -x-step 1.0 -x-base 10.0 -x-expression pow(BASE,I) -sample-size 100.0 -traversal ROW-WISE -log-file "C:\\Pr	0.963	0.067	0.964	0.962	0.9	Weighted Avg.	96.28	3.7209	0	0	0	2	l	d	=	##
Scheme: RandomizableFilteredClassifier+IBk weka.classifiers.meta.GridSearch -E CC -v-property seed -v-min -3.0 -v-max 3.0 -v-step 1.0 -v-base 10.0 -v-expression pow(BASE,I) -x-property debug -x-min -3.0 -x-max 3.0 -x-step 1.0 -x-base 10.0 -x-expression pow(BASE,I) -sample-size 100.0 -traversal ROW	1	0.089	0.97	0.985	0.9	1			a	b	c	d	<--	classified	as	
Scheme: weka.classifiers.meta.GridSearch -E CC -v-property seed -v-min -3.0 -v-max 3.0 -v-step 1.0 -v-base 10.0 -v-expression pow(BASE,I) -x-property debug -x-min -3.0 -x-max 3.0 -x-step 1.0 -x-base 10.0 -x-expression pow(BASE,I) -sample-size 100.0 -traversal ROW-WISE -log-file "C:\\Program Files\\	0.917	0	1	0.957	1	2			##	0	0	0	l	a	=	1
Scheme: weka.classifiers.meta.GridSearch -E CC -v-property seed -v-min -3.0 -v-max 3.0 -v-step 1.0 -v-base 10.0 -v-expression pow(BASE,I) -x-property debug -x-min -3.0 -x-max 3.0 -x-step 1.0 -x-base 10.0 -x-expression pow(BASE,I) -sample-size 100.0 -traversal ROW-WISE -log-file "C:\\Program Files\\	0.889	0	1	0.941	0.9	3			3	33	0	0	l	b	=	2
Scheme: weka.classifiers.meta.GridSearch -E CC -v-property seed -v-min -3.0 -v-max 3.0 -v-step 1.0 -v-base 10.0 -v-expression pow(BASE,I) -x-property debug -x-min -3.0 -x-max 3.0 -x-step 1.0 -x-base 10.0 -x-expression pow(BASE,I) -sample-size 100.0 -traversal ROW-WISE -log-file "C:\\Program Files\\	1	0	1	1	1	999			2	0	16	0	l	c	=	3
Scheme: weka.classifiers.meta.GridSearch -E CC -v-property seed -v-min -3.0 -v-max 3.0 -v-step 1.0 -v-base 10.0 -v-expression pow(BASE,I) -x-property debug -x-min -3.0 -x-max 3.0 -x-step 1.0 -x-base 10.0 -x-expression pow(BASE,I) -sample-size 100.0 -traversal ROW-WISE -log-file "C:\\Program Files\\	0.977	0.066	0.977	0.976	0.9	Weighted Avg.	97.67	2.3256	0	0	0	2	l	d	=	##
